# Fluidity of gender identity induced by illusory body-sex change

**DOI:** 10.1038/s41598-020-71467-z

**Published:** 2020-09-01

**Authors:** Pawel Tacikowski, Jens Fust, H. Henrik Ehrsson

**Affiliations:** 1 Department of Neuroscience, Karolinska Institute, Stockholm, Sweden; 2 Department of Neurosurgery, Univeristy of California Los Angeles, Los Angeles, USA; 3 Department of Clinical Neuroscience, Karolinska Institute, Stockholm, Sweden

**Keywords:** Cognitive neuroscience, Human behaviour

## Abstract

Gender identity is a collection of thoughts and feelings about one’s own gender, which may or may not correspond to the sex assigned at birth. How this sense is linked to the perception of one’s own masculine or feminine body remains unclear. Here, in a series of three behavioral experiments conducted on a large group of control volunteers (N = 140), we show that a perceptual illusion of having the opposite-sex body is associated with a shift toward a more balanced identification with both genders and less gender-stereotypical beliefs about own personality characteristics, as indicated by subjective reports and implicit behavioral measures. These findings demonstrate that the ongoing perception of one’s own body affects the sense of one’s own gender in a dynamic, robust, and automatic manner.

## Introduction

Gender identity is a collection of thoughts and feelings about one’s own gender, which may or may not correspond to the sex assigned at birth^1–5^. This multifaceted, subjective sense of being male, female, both, or neither occurs in our conscious self-awareness, but the associated perceptions and beliefs can also be largely implicit^3–5^. In the past, gender identity was conceptualized as a male–female dichotomy; however, current theories consistently postulate that gender identity is a spectrum of associations with both genders^1,3–6^. There is also a general consensus in the field that gender identity is determined by multiple factors, such as person’s genes, hormones, patterns of behaviors, or social interactions^4–8^; and that the sense of own gender (e.g., “I’m male”) is closely linked to one’s beliefs about males and females in general (e.g., “males are competitive”), as well as to the associated beliefs about own personality (“I am competitive”)^1,3,6^. The specific content of such beliefs and their strength contribute to what it means for a given person to be male or female in a given sociocultural context, which in some cases hinders the realization of one’s full personal or professional potential. Although gender identity has a profound impact on our lives, little is known about how this sense is formed or maintained. A better understanding of the neurocognitive mechanisms of gender identity is also important in the context of gender dysphoria (DSM-5^9^; gender incongruence ICD-11^10^), which is characterized by the prolonged and clinically relevant distress that some transgender individuals experience due to inconsistency between their sex assigned at birth and their subjective sense of gender.

Various observations suggest that gender identity and the perception of one’s own body are tightly connected. For example, people with gender dysphoria (see above) often avoid looking in the mirror, hide their bodies under loose-fitting clothes, and seek hormonal and/or surgical procedures to adjust their physical appearance to meet their subjective sense of own gender^6,11,12^. Moreover, among individuals whose gender identity matches their sex assigned at birth, mastectomy and androgen deprivation cancer therapies, which both involve changes to one’s feminine or masculine bodily characteristics, are often related to a gender identity crisis^13,14^. There are also data suggesting that the mental representation of one’s own body is altered in transgender individuals^15^ and that the brain regions involved in this representation are anatomically and functionally different in this group compared to controls^16–23^. However, the link between own body perception and gender identity remains poorly understood from a behavioral experimental perspective, and we do not know whether, and if so how, the perceived sex of own body influences the sense of own gender in nontransgender individuals.

The full-body ownership illusion^24^ is a powerful experimental tool for manipulating the perception of one’s own body^25–30^. During this illusion, the participants wear head-mounted displays (HMDs) and observe a stranger’s body from a first-person perspective. The stranger’s body is continuously stroked with a stick or a brush, and the experimenter applies synchronous touches on the corresponding parts of the participant’s body, which is out of view. Synchronous visuotactile stimulation induces a feeling that the stranger’s body is one’s own, whereas asynchronous stimulation breaks the illusion and serves as a well-matched control condition^24,31–33^. The full-body ownership illusion, similar to the rubber hand illusion involving a single limb^34–37^, occurs when visual, tactile, proprioceptive, and other sensory signals from the body are combined at the central level into a coherent multisensory representation of one’s own body^24–26,30^. Body ownership illusions involving limbs^37^ and full bodies^31,33,38^ are related to increased neural activity in regions of the frontal and parietal association cortices that are related to multisensory integration, such as the premotor and intraparietal cortices. Because these brain regions contain trimodal neurons that integrate visual, tactile, and proprioceptive signals and because body illusions closely follow the temporal and spatial constraints of multisensory integration, it has been proposed that combining bodily signals from different modalities is a key mechanism for attributing ownership to our bodies^25–30^. The full-body illusion has been replicated in numerous studies^24,31–33,39–45^, even with bodies of a sex opposite to that of the participants^24,43^, but the cognitive consequences of this transient physical sex change on gender identity have not been assessed.

Here, we conducted three within-subject behavioral experiments on a total of one hundred forty naïve control volunteers to investigate a possible dynamic relationship between the perception of own body and the sense of own gender. In all three experiments, we induced the “body-sex-change illusion”, which is analogous to the standard full-body illusion (see earlier), but in the HMDs, the participants observe the opposite-sex stranger’s body (Movies S1 and S2). Thus, we aimed to experimentally manipulate how the participants perceived the secondary sex characteristics of their own bodies to measure what outcome this manipulation has on different aspects of gender identity. Specifically, in Experiment I, we asked the participants to rate how masculine or feminine they felt after the body-sex-change illusion to assess the subjective and conscious facets of gender identity. Explicit methods such as the one above provide information about participants’ phenomenological experience, but ideally, they should be combined with objective tests to provide more conclusive results. Therefore, in Experiment II, we used a well-controlled behavioral method—the Implicit Association Test (IAT)—to measure the cognitive and implicit aspects of gender identity; this test is largely immune to conscious strategies^46^ and has been validated for gender identity research in control^47,48^ as well as in transgender individuals^48^. Finally, in Experiment III, we tested whether the perception of own body affects gender-related beliefs about own personality (see earlier) by asking the participants to rate after the illusion how much different traits, stereotypically related to males and females, pertain to their own personality. Applying such different measurements aimed to capture the multifaceted character of the sense of own gender (see earlier), while using continuous scales in all experiments addressed gender identity as a spectrum (see earlier). We hypothesized that if own body perception dynamically shapes gender identity, then even a brief transformation of one’s own perceived physical sex during the body-sex-change illusion should shift different aspects of gender identity toward the opposite gender.

## Results

**Figure 1:**
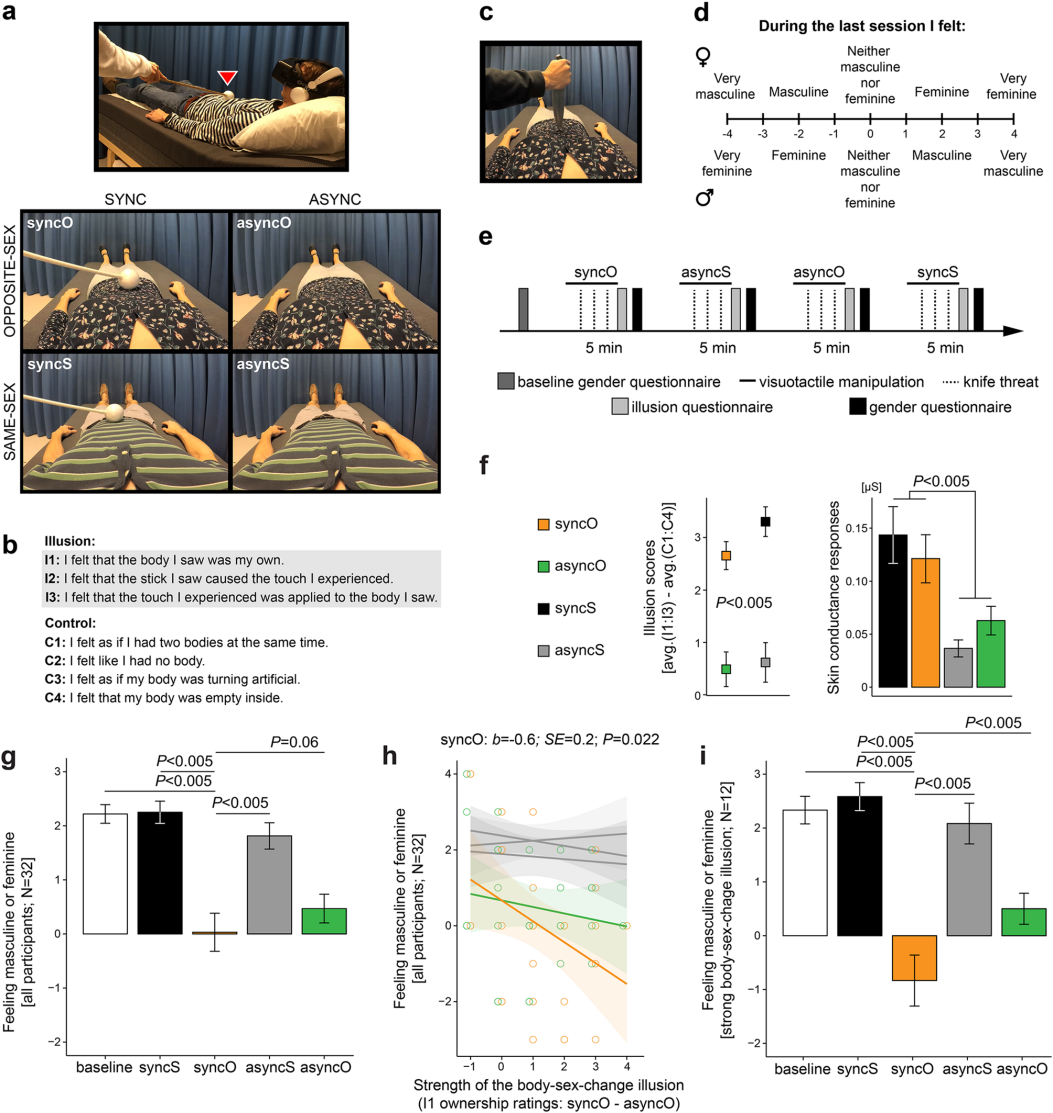
Perceptual illusion of having the opposite-sex body modulated the subjective experience of feeling masculine or feminine (Experiment I). (**a**) The participants (N = 32; 15 females) lay on a bed and wore a head-mounted display in which a body of an unknown male or female was shown from a first-person perspective (the participant’s real body was out of view). Video frames illustrate all four conditions for a male participant (top picture). For a female participant, the videos from the lower and upper rows would be swapped. In the synchronous conditions, touches applied to the participant and touches applied to the stranger’s body were matched (see the red triangle), whereas in the asynchronous conditions, touches applied to the participants were delayed by 1 s. We expected to induce the body-sex-change illusion specifically in the syncO condition, and the other conditions served as controls. (**b**) After each condition, the participants rated illusion (I1:I3) and control (C1:C4) statements on a 7-point scale (− 3—“strongly disagree”; + 3— “strongly agree”). The illusion statements assessed the feeling that the stranger’s body is one’s own, whereas the control statements controlled for any potential effects of suggestibility or task compliance. (**c**) Genuine ownership of the stranger’s body should be associated with increased physiological stress responses of the participant when the stranger’s body is physically threatened. Thus, we measured the participants’ skin conductance responses elicited by brief “knife threat” events that occurred in the videos. (**d**) Before the experiment (baseline) and after each condition, the participants rated how feminine or masculine they felt. The upper row shows scale assignment for female participants and the lower row for males. (**e**) The order of conditions was counterbalanced across the participants, and the whole experiment lasted ~ 30 min. (**f**) The illusion ratings and the magnitude of skin conductance responses were significantly higher in the synchronous than in the asynchronous conditions, which shows that the full-body ownership illusion was elicited as expected. (**g**) During syncO, the female participants indicated feeling less feminine, and the male participants indicated feeling less masculine than during other conditions. (**h**) Strong illusory ownership of the opposite-sex body was related to a significant shift toward the opposite gender, specifically in syncO. For clarity of display, only ratings from syncO and asyncO are shown; syncS, asyncS, and baseline are colored in gray for comparison. (**i**) The participants who experienced a strong body-sex-change illusion (above-median I1 ownership ratings: syncO–asyncO; N = 12) indicated feeling more masculine (females) or more feminine (males) during syncO than during other conditions. Plots show means ± SE.

Experiment I tested whether the perception of one’s own body dynamically shapes one’s subjective feeling of masculinity/femininity. The experiment comprised a two-by-two factorial design with four conditions (Fig. 1a): “synchronous opposite sex” (syncO), “synchronous same sex” (syncS), “asynchronous opposite sex” (asyncO), and “asynchronous same sex” (asyncS). This design allowed us to manipulate the sex-related characteristics of the perceived bodily self in the body-sex-change illusion condition (syncO) while controlling for potential confounding factors related to experiencing a full-body ownership illusion itself (syncS) or cognitive biases due to simply looking at a male or female body (asyncS, asyncO). We measured the illusion psychometrically by asking the participants to rate their subjective experience of owning the stranger’s body (Fig. 1b) and objectively by recording the participants’ physiological fear reactions (skin conductance responses) when the stranger’s body was physically threatened with a knife (Fig. 1c). Both of these illusion measures should be higher during the synchronous than during the asynchronous conditions^24,32,38,39,42^. Importantly, before experiencing any body perception manipulation (baseline) and after every full-body illusion condition, the participants rated how masculine or feminine they felt (Fig. 1d,e).

The full-body ownership illusion was induced as expected, that is, “illusion scores” [illusion questionnaire ratings: (I1 + I2 + I3)/3 + (C1 + C2 + C3 + C4)/4] were higher in the synchronous than in the asynchronous conditions, and knife threats during the synchronous conditions triggered stronger skin conductance responses than knife threats during the asynchronous conditions (Fig. 1f; Table S2; main effect of synchrony; illusion scores: *F*_1,32_ = 64.48; *P* < 0.005; skin conductance: *F*_1,27_ = 10.98; *P* < 0.005; two-sided; N = 32). With regard to our main hypothesis, we found that during syncO, female participants indicated feeling significantly less feminine and male participants significantly less masculine than during the baseline, syncS, and asyncS control conditions; the difference between syncO and asyncO showed a significant trend in the hypothesized direction (Fig. 1g; Tables S2 and S3). Importantly, the shift toward the opposite gender, specifically in the syncO condition, was enhanced by the illusory ownership of the opposite-sex body (Fig. 1h; Tables S2 and S3; synchrony × body × ownership: *F*_1,32_ = 8.05; *P* = 0.008; main effect of ownership in syncO: *b* = − 0.6; *SE* = 0.2; *t*_30_ = − 2.29; *P* = 0.022; two-sided; N = 32). Please note that “ownership” in the analysis above corresponds to I1 questionnaire ratings: syncO—asyncO (one value per participant). Moreover, we found that the participants who experienced a strong body-sex-change illusion (N = 12; median-split; see “Materials and methods”) indicated feeling more like the opposite gender in syncO compared to the other conditions (Fig. 1i; Tables S2 and S3). Overall, Experiment I shows that the ongoing perception of one’s own body dynamically updates one’s subjective feelings of masculinity or femininity.

**Figure 2:**
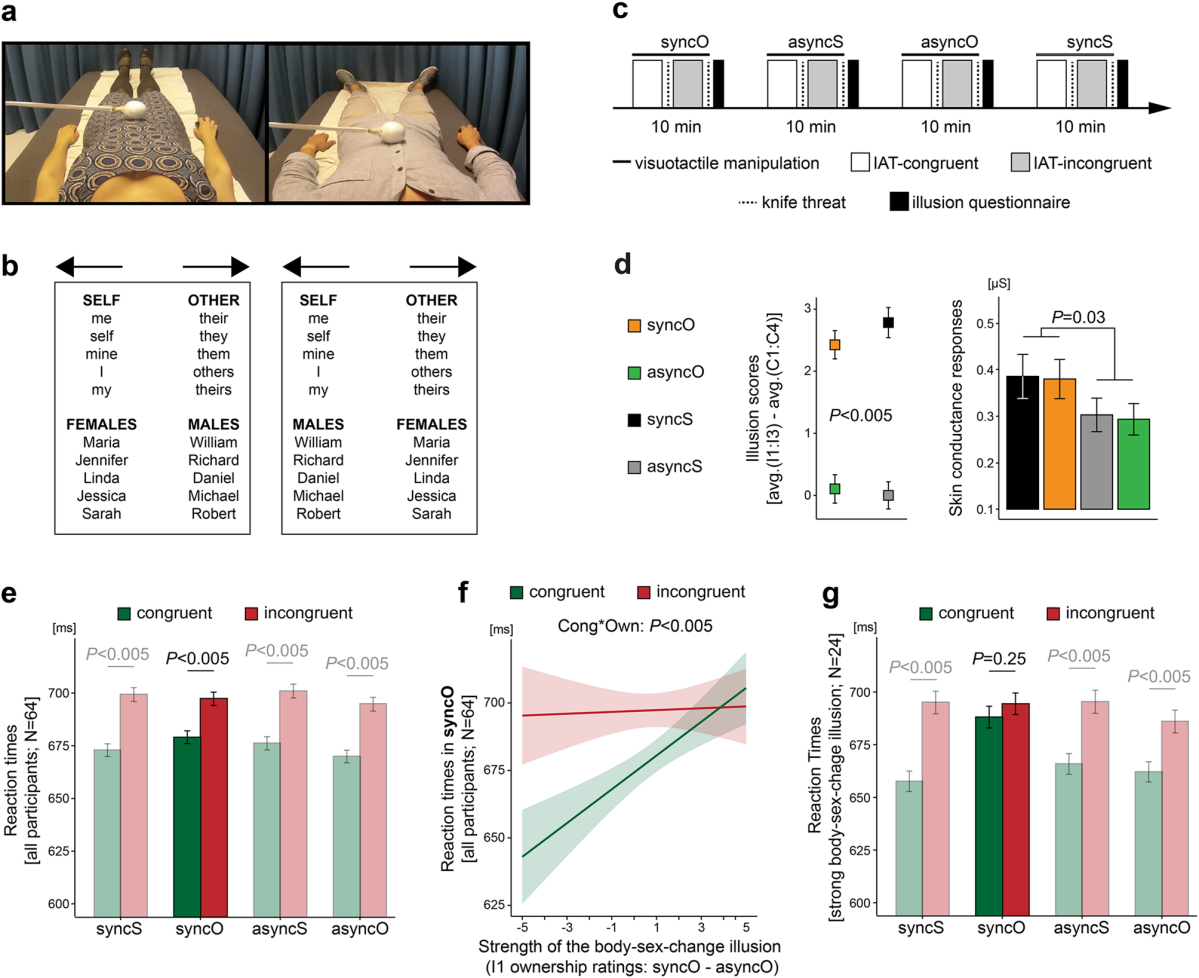
The body-sex-change illusion balanced implicit associations between the self and both genders (Experiment II). (**a**) This experiment (N = 64; 32 females) comprised the same four conditions as Experiment I (Fig. 1a), but we used recordings of a different male and female body to enhance the generalizability of our findings. (**b**) The left panel is a schematic representation of the congruent IAT block for the female participants (incongruent for males), as words from the *self* and *female* categories are assigned to the same response category (left arrow). The right panel shows the IAT block that is incongruent for the female participants and congruent for males. Please note that exactly the same words are used in both blocks, only the instructions (key assignment) are different. (**c**) During each condition, the participants completed one full IAT (two blocks). The condition order and block order were counterbalanced across participants. The whole experiment lasted ~ 60 min. (**d**) The body-sex-change illusion was successfully induced, as shown by questionnaire data and the magnitude of threat-evoked skin conductance responses. (**e**) In all conditions, reaction times were significantly shorter in the congruent than in the incongruent IAT blocks, which shows that it was generally easier for the participants to associate themselves with the gender consistent with their sex. (**f**) Strong illusory ownership of the opposite-sex body was related to the balancing of implicit associations between the self and both genders, specifically in syncO. For clarity of display, individual data points are not shown (n = 7,290). (**g**) The participants who experienced a strong body-sex-change illusion (above-median I1 ownership ratings: syncO–asyncO; N = 24) responded similarly quickly in the incongruent and congruent IAT blocks during syncO. Bar plots show means ± SE.

Experiment II tested whether the perceived sex of one’s own body also modulates *implicit* associations between oneself and gender categories. This experiment had the same two-by-two factorial design as Experiment I (Fig. 1a), but this time, gender identity was measured with the IAT^47,48^. During this test, the participants heard words belonging to four semantic categories (*male*, *female*, *self*, or *other*) and sorted these words into just two response categories. In one block, the participants responded with the same key to words from the *self* and *female* categories, which made this block congruent for females and incongruent for males. In the other block, the participants responded with the same key to words from the *self* and *male* categories, which made this block incongruent for females and congruent for males (Fig. 2b). Faster responses in the congruent block than in the incongruent block (i.e., congruent block being cognitively less demanding) indicate that a given person associates with the gender that is consistent with his/her sex. In turn, longer reaction times in the congruent block suggest an inclination toward the opposite gender, whereas similar responses in both blocks suggest a balanced gender identity. Thus, the IAT provides a fine-grained behavioral proxy of where a person is located on a gender identity spectrum (see “Introduction”). The participants performed the IAT four times, once *during* each condition, which allowed us to track changes in implicit gender identification across different embodiment contexts (Fig. 2c).

The body-sex-change illusion was also successfully induced in Experiment II, as demonstrated by the questionnaire and skin conductance data (Fig. 2d; Table S4; main effect of synchrony; illusion scores: *F*_1,64_ = 125.65; *P* < 0.005; skin conductance: *F*_1,60_ = 4.97; *P* = 0.03; two-sided; N = 64). In all conditions, it was easier for the participants to associate themselves with the gender consistent with their sex, as indicated by shorter reaction times in the congruent than in the incongruent blocks (Fig. 2e; Tables S4 and S5). More importantly, however, strong illusory ownership of the opposite-sex body was related to a reduced difference between the incongruent and congruent blocks specifically in syncO, which shows that the illusion balanced the strength of implicit associations between the self and both genders (Fig. 2f; Table S4; synchrony × body × congruence × ownership: *F*_1,28878_ = 17.03; *P* < 0.005; congruence × ownership in syncO: *F*_1,7207_ = 9.37; *P* < 0.005; two-sided; N = 64). Furthermore, the participants who experienced strong illusory ownership of the opposite-sex body (N = 24; median-split; see “Materials and methods”) had similar reaction times in the congruent and incongruent IAT blocks during syncO (Fig. 2g; Tables S4 and S5). Thus, the main finding of Experiment II is that the moment-to-moment perception of one’s own body balances the strength of implicit associations between the self and both genders.

**Figure 3:**
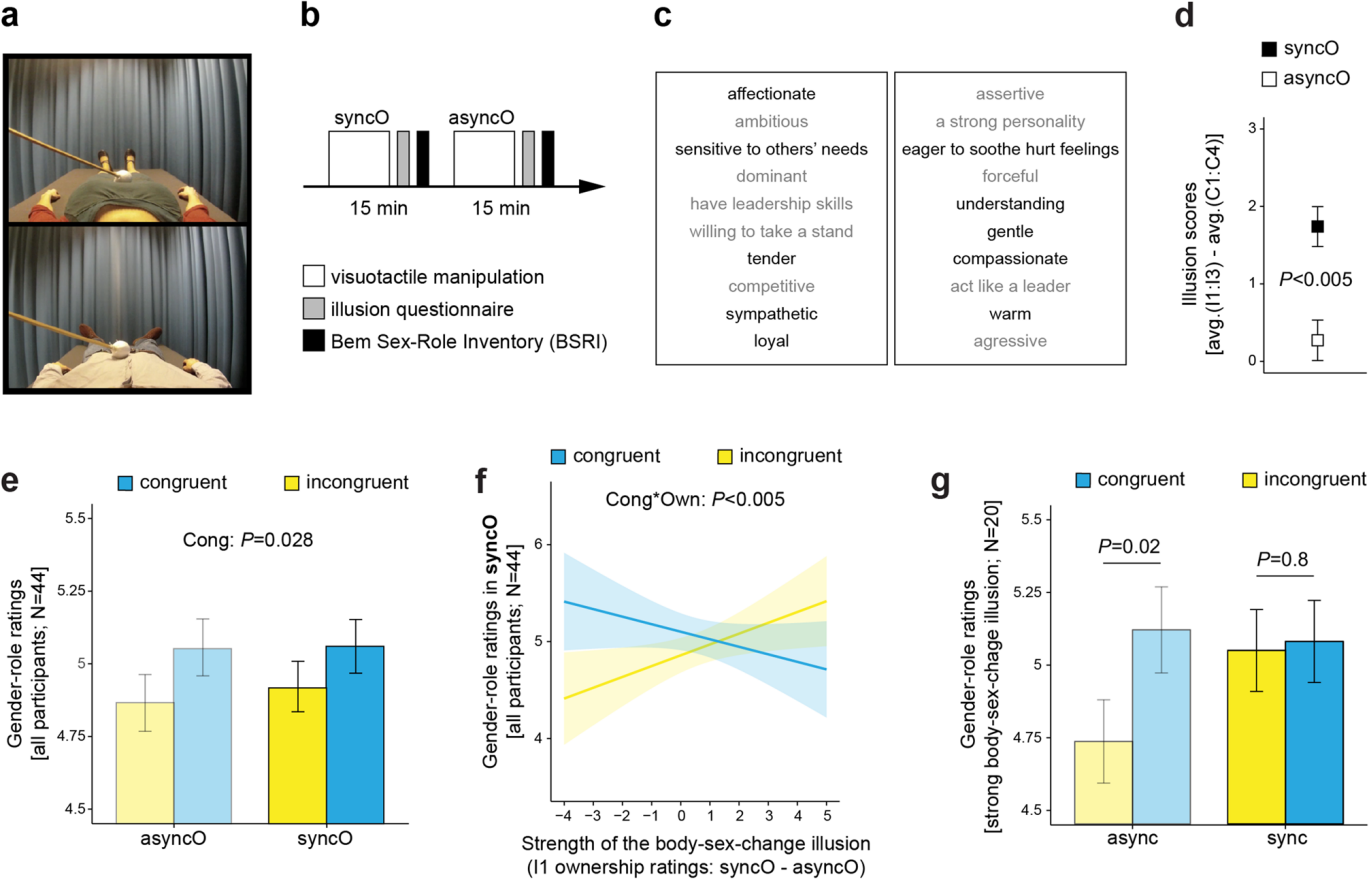
Illusory ownership of the opposite-sex body was associated with less gender-stereotypical beliefs about one’s own personality traits (Experiment III). (**a**) Frames from the videos used in this experiment (N = 44; 22 females). (**b**) The experiment consisted of two conditions (syncO and asyncO); thus, in the head-mounted display, the female participants always observed a male body and the male participants a female body that was stroked either synchronously or asynchronously with regard to touches delivered to the participants. The condition order was counterbalanced across the participants, and the whole experiment took ~ 45 min. (**c**) After each condition, the participants rated how well each personality characteristic describes the self (1—“not at all”; 7—“very much”). Each BSRI sublist contained five traits stereotypically related to males (gray) and five traits stereotypically related to females (black). (**d**) The illusion ratings were significantly higher in the syncO condition than in the asyncO condition, which demonstrates that the body-sex-change illusion was efficiently induced. (**e**) Stereotype-congruent personality traits were generally rated higher than stereotype-incongruent traits. (**f**) Strong illusory ownership of the opposite-sex body was associated with less gender-stereotypical beliefs about own personality traits, specifically in the syncO condition. For clarity of display, individual data points are not shown (n = 434). (**g**) The participants who experienced a strong body-sex-change illusion (above-median I1 ownership ratings: syncO–asyncO; N = 20) rated stereotype-congruent and stereotype-incongruent traits similarly high, specifically in the syncO condition. Bar plots show means ± SE.

Experiment III investigated whether the illusion-induced fluidity of gender identity is generalized to gender-related beliefs about one’s own personality (see “Introduction”). This experiment consisted of two conditions (syncO and asyncO); thus, in the HMDs, female participants always observed a male body, and male participants always observed a female body (Fig. 3a,b). After each condition, the participants filled out a short version of the Bem Sex-Role Inventory (BSRI)^49,50^ that contained personality characteristics stereotypically associated with males and females (Fig. 3b,c). The participants’ task was to rate how much they thought each trait refers to their own personality. We found that the body-sex-change illusion was efficiently induced in Experiment III as well (Fig. 3d; Table S6; main effect of synchrony; illusion scores: *F*_1,44_ = 35.88; *P* < 0.005; two-sided; N = 44). Ratings of stereotype-congruent traits were generally higher than ratings of stereotype-incongruent traits (Fig. 3e; Tables S6 and S7). Crucially, however, this stereotypical tendency was significantly reduced in the syncO condition by the illusory ownership of the opposite-sex body (Fig. 3f; Tables S6 and S7; synchrony × congruence × ownership: *F*_1,759_ = 5.6; *P* = 0.018; congruence × ownership in syncO: *F*_1,374_ = 13.46; *P* < 0.005; two-sided; N = 44). The participants who experienced a strong body-sex-change illusion (N = 20; median-split; see “Materials and methods”) rated stereotype-congruent and stereotype-incongruent traits during syncO similarly high (Fig. 3g; Table S7). These findings show that the perception of one’s own masculine or feminine physical characteristics flexibly updates gender-stereotypical beliefs about one’s own personality.

Finally, to assess the overall robustness of the relationship between own body perception and gender identity, we performed a post hoc meta-analysis of the data from all three experiments combined. We found that strong illusory ownership of the opposite-sex body in syncO was related to increased updating of the sense of own gender (Fig. S1; *ρ*_138_ = 0.24; *P* < 0.005; Spearman correlation; two-tailed). Control analyses showed that the male and female participants experienced the body-sex-change illusion equally strongly and that there was no consistent significant relationship between the illusion strength and the participants’ age or baseline masculinity/femininity ratings (Fig. S2; for analogous evidence regarding syncS, see Fig. S3). Moreover, the degree of gender identity updating did not significantly differ between males and females and was not significantly related to the participants’ age or baseline masculinity/femininity ratings (Fig. S4). These results are in line with the notion that the full-body illusion is a robust perceptual phenomenon that generally is not affected by high-level cognitive or emotional processes^25,30^, which validates the current illusion-based approach to dynamically changing the perceptual basis of the bodily self in a mixed group of male and female subjects.

## Discussion

The present study used the body-sex-change illusion to experimentally investigate the link between own body perception and gender identity. We found that even a brief transformation of one’s own perceived bodily sex dynamically updated the subjective, implicit, and personality-related aspects of the sense of own gender and made these aspects more balanced across male and female categories. This main finding was consistent across three separate experiments conducted on a large group of control volunteers, with the use of subjective and objective behavioral measures. The fluidity of gender identity that we report here extends previous knowledge by demonstrating that the link between own body perception and the sense of own gender is dynamic, robust, and direct. It is dynamic because the effects that we detected occurred after several minutes of the body-sex-change illusion, it is robust because these effects were present at explicit and implicit levels, and it is direct because the changes in gender identity precisely followed our experimental manipulation of perceived own body sex.

By highlighting the role of own body perception in the shaping of the sense of own gender, this study adds a new perspective to existing theories of gender identity development. Specifically, it has been previously proposed that during their first year of life, infants construct presymbolic, perceptual, and unconscious representations of gender, based on patterns of maternal and paternal interactive styles; their touch, activity levels, timbre of voice, affective reactions, etc.^4,5^. Around the same age, babies can also detect synchronous visuotactile and synchronous visuomotor information related to their body^51–54^, which suggests that they have already developed a basic multisensory representation of their own body that continues to mature throughout childhood^55^. Thus, it is possible that during direct interactions with their caregivers, infants experience various degrees of sensory alignment between their own body representation and the perceptual representations of gender; this alignment might be a foundation for what older children and adolescents refine into a conscious sense of own gender^4,5^. The present findings fit well with the above idea and offer important new insights by showing that the moment-to-moment perception of one’s own body continues to affect gender identity even in adult participants.

The fluidity of gender identity that we demonstrate here does not deny that most people experience a stable sense of own gender. Instead, our findings indicate that a change is *possible* if a sufficient modification of own body representation occurs. Thus, the present study is in line with the general notion that gender identity is a “softly assembled, self-organizing system” that involves dynamic coupling between relevant biological, psychological, and sociocultural factors, such as a person’s hormonal and anatomical status, thoughts and feelings about his or her own gender, or perceived societal norms; when all these factors cohere tightly, gender identity remains stable, but when coherence is poor, gender identity is updated accordingly^4,5,8^. What current results add to this perspective is experimental support that the perception of own secondary sex characteristics is an integral part of the gender identity construction process that can considerably perturb the sense of own gender in nontransgender adults.

A thorough reader might ask how, if the perception of one’s own body is so critical for gender identity, these two aspects can remain in conflict for a prolonged period of time in transgender individuals. First, our results should not be treated as evidence that perceived bodily sex is the *only* factor that shapes the sense of own gender; this sense is a complex phenomenon that is constructed from multiple factors (see “Introduction”). Second, some characteristics of gender dysphoria, such as avoiding looking in the mirror or hiding one’s body under loose-fitting clothes^11,12^, suggest that these individuals might actively suppress the link between their own body perception and their subjective sense of gender. Our results contribute to the discussion about the mechanisms of gender identity by suggesting that there is a continuous bottom-up influence from the perceptual body representation on the cognitive, conceptual, and possibly affective representations of gender identity in terms of the body’s secondary sexual characteristics. Future studies should address the important question of how transgender people, with and without gender dysphoria, update their sense of own gender during the body-sex-change illusion and whether the illusion could partly alleviate distress by reducing the incongruence between the body and subjective gender.

Another key finding of the present study is that the body-sex-change illusion reduced gender-stereotypical beliefs about own personality. This result supports the claim that gender identification (e.g., “I’m a male”), gender stereotypes (e.g., “Males are competitive”), and gender-stereotypical beliefs about one’s own personality (e.g., “I’m competitive”) are connected with each other^1,3,6^, so that a change in one aspect (gender identification), due to the body-sex-change illusion, affects the other aspects (stereotypical self-beliefs). It is worth mentioning that existing programs against gender discrimination, such as media campaigns or educational workshops, mainly target explicit manifestations of gender stereotypes^56^. However, people are often unaware that their way of thinking is biased, and thus, they cannot deliberately change it. Body-oriented techniques, similar to the one used here, could possibly overcome this limitation and target more covert aspects of gender discrimination. Future research is needed to validate this approach.

Previous studies have shown that different versions of the full-body ownership illusion have various cognitive, emotional, and behavioral consequences. For example, attitudes toward other people change after illusory ownership of their bodies^29^, emotional feelings of social fear^42^ and body dissatisfaction^32,33^ can be modulated by the full-body ownership illusion, and the encoding of episodic memories depends on the embodied first-person perspective^57^. Even beliefs about own personality characteristics^58^, the recognition of one’s own face^59–61^, the style of one’s own behavior^62^, and implicit associations with the past-self^45^ are flexibly adjusted based on the ongoing perception of one’s own body. With regard to gender, it has been shown that it is possible to induce the body-sex-change illusion^24,43^ and that female participants who looked at male avatars from a first-person perspective improved their working memory performance during a stereotype-threatening situation^63^; however, the latter finding needs to be interpreted with caution, as there was no conclusive evidence that the participants felt ownership of the avatar’s body. Our study extends the above literature in three ways: first, by showing that even the supposedly most stable aspects of the psychological sense of self, that is, gender identity, are dynamically updated based on the ongoing perception of one’s own body; second, by demonstrating that this updating affects both implicit and explicit beliefs about the self; and third, by clarifying that the illusory ownership of another person’s body not only modifies attitudes toward that person or toward a social group that he or she is a member of but also modifies beliefs about the self.

With regard to the cognitive mechanisms behind the body-related flexibility of self-concept, there are several possible explanations. Embodied cognition theories propose that all concepts are grounded in sensorimotor and situated representations^64^; thus, a change in own body representation, for example, during a full-body ownership illusion, affects conceptual knowledge about the self. In turn, predictive processing theories suggest that if the low-level perceptual representation of one’s own body creates a conflict further up in the processing hierarchy, then this conflict is resolved by updating higher-order beliefs about oneself^28,65^. Other authors proposed that illusory ownership of someone else’s body (1) involves making inferences about own attitudes, e.g., “I am polite, because the person whose body I have is polite”^62^; (2) that the illusion allows new associations to be formed within the “self-image network”^66^; (3) that “owning” another person’s body makes knowledge about that person, or about a social group that this person belongs to, more accessible (i.e., primed) in the conceptual knowledge system^67^; or (4) that body experiences of this kind increase the perceived physical similarity between the self and the other, which consequently increases the perceived conceptual similarity between the two^29^. What the present study adds to this complex discussion is the demonstration that gender identity updating is not a result of deliberate inference, as the effect occurred for implicit associations measured by the IAT; and this updating could not simply be explained by conceptual priming, because the semantic category of the opposite gender was likely “activated” even by looking at the opposite sex body during asyncO. Moreover, our results suggest that creating new associations within the self-image network is not the only mechanism involved in the updating of self-concept because, at the implicit level, the body-sex-change illusion mainly *weakened* associations between the self and the preferred gender category (i.e., lengthening reaction times in the congruent IAT block; Fig. 2f; Table S5). Thus, the perceived bodily-sex-change possibly increased a cognitive conflict within the existing beliefs about oneself at the implicit level, which in turn was compensated by revising self-beliefs with new information at the explicit level (i.e., increasing ratings of stereotype-incongruent traits; Fig. 3f; Table S7). Future studies should determine whether the body-related flexibility of self-concept involves different cognitive mechanisms depending on the degree of conscious awareness.

We speculate that at the neural level, the fundamental interplay between the perception of one’s own body and gender identity is implemented by functional interactions between the multisensory frontoparietal areas that represent the bodily self^30,31,37^, on the one hand, and the medial prefrontal regions that are involved in the self-concept representation^68,69^, affective body representations in the insula and anterior cingulate cortex^33^, and higher-order visual representation of the body in the lateral occipital cortex^70,71^, on the other. Multisensory representations in the posterior parietal cortex may be particularly important in this respect, as this region is sensitive to the perceived size and shape of one’s own body^33,72^, including waist size^72^, which is likely to be important for the identification of the body’s sex based on secondary sex characteristics. Notably, the pattern of resting-state connectivity in the posterior parietal cortex is different in transgender individuals compared to age-matched controls^21^, and a recent study reported that individuals with gender dysphoria display greater cortical thickness of the anterior cingulate cortex and lateral occipital cortex than controls^23^. Interestingly, the lateral occipital cortex, which includes the extrastriate body area—a higher-order visual area that is involved in the processing of images of human body parts^73^—shows increased activation during body ownership illusions^33,38,70,71^. Future neuroimaging studies could use the present body-sex-change illusion to perturb the sense of gender identity experimentally and investigate how the patterns of activity and functional connectivity within the above fronto-parieto-occipital networks change accordingly.

It is noteworthy that our findings are mainly related to balancing the identification with both genders rather than to a “full switch” to the opposite gender. This could be because the perception of one’s own body is not potent enough to completely override the existing sense of own gender or because the body-sex-change illusion in the present study was not induced for long enough. Future studies are needed to reveal the extent to which gender identity could change as a result of modified body representation and the persistence of such changes over time. Another methodological aspect that is noteworthy is that the body-induced fluidity of gender identity showed relatively large interindividual differences. This variability was related mainly to how vividly the participants experienced the body-sex-change illusion, which of course makes sense because if there was no change in the representation of own body, then there was no reason to update one’s gender identity. Individual differences in the strength of body ownership illusions are most likely related to how brains integrate visual, tactile, and proprioceptive signals^30,74^ and depend on the relative weights assigned to different sensory channels, as well as prior knowledge that varies across subjects^34,75^. For example, if more weight is given to vision than to proprioception, the illusion should be stronger, and vice versa. Based on our data, we can conclude that variability in the illusion strength was not significantly related to the participants’ sex, age, or baseline feelings of masculinity/femininity (Figs. S2 and S3). Finally, it is worth mentioning that our within-subject experimental design allowed us to demonstrate a particularly strong case of gender identity flexibility that occurred for the *same* participants across different body perception contexts.

In sum, the present study shows that there exists a dynamic and automatic link between the perception of own body and different aspects of the sense of own gender. This main finding has important bearings on neurocognitive models of gender identity, as well as on clinical psychology and psychiatry. Moreover, the body-sex-change illusion that we report here allows for a manipulation of gender identification in nontransgender participants, which offers an unprecedented opportunity to investigate the sense of own gender in a controlled experimental setting. Importantly, people with gender dysphoria who consider surgical and hormonal procedures to adjust their physical appearance to match their gender identity could perhaps benefit from future iterations of the body-sex-change illusion, which combined with virtual reality and 3D body scanners might alleviate distress and allow these individuals to somewhat experience their own “new body” before undergoing more permanent procedures.

## Materials and methods

All participants provided written informed consent before the start of each experiment. The Regional Ethics Review Board of Stockholm approved the studies. All methods were performed in accordance with the approved guidelines. The inclusion criteria were as follows: (1) age between 18 and 65 years old; (2) no history of severe psychiatric illness or neurological disorder; (3) normal or corrected-to-normal vision and hearing; (4) not wearing glasses during the experiment; and (5) understanding English (see below). These criteria were assessed during an initial interview. Sample sizes were based on similar previous studies (see “Introduction”) and our counterbalancing schemes. Data collection was finalized when the planned number of participants was reached. At the end of each experiment, the participants were debriefed and received compensation. All measures that were used are reported in the manuscript. Because the participants were of different nationalities, all experiments were conducted in English; the participants followed instructions without problems. The stroking procedure in Experiment I was performed by P.T., and in Experiments II and III, it was performed by J.F.

### Experiment I

#### Participants

Thirty-three naïve adults participated (age: 25 ± 4; 4 left-handed; 15 females). Data from one participant were excluded due to a procedural error (same condition repeated twice).

#### Procedure

The participants first rated how masculine or feminine they felt before experiencing any body perception manipulation (baseline; Fig. 1d). The main experiment consisted of four conditions: “synchronous opposite sex” (syncO), “synchronous same sex,” (syncS), “asynchronous opposite sex,” (asyncO), and “asynchronous same sex” (asyncS). Each condition lasted 3.5 min. During each condition, the participants lay on a bed with their heads tilted forward (~ 45°) and wore a head-mounted display (HMD; Oculus Rift Development Kit 2, Oculus VR, Menlo Park, CA, USA) so that they could not see their actual body. In the HMD, the participants watched prerecorded 3D videos of a stranger’s body, male or female, that was shown from a first-person perspective. The stranger’s body was continuously stroked on the thighs and abdomen, and the experimenter delivered synchronous (syncO, syncS) or asynchronous (1 s delayed; asyncO, asyncS) touches on the corresponding parts of the participant’s body (Fig. 1a). During each condition, there were three “knife threats” that occurred 1, 2, and 3 min after the beginning of each video (Fig. 1c,e). After each condition, the participants took off the HMD, filled out the illusion questionnaire (Fig. 1b) and rated how masculine or feminine they felt during the preceding session (Fig. 1d). The order of conditions was counterbalanced across the participants, and the whole experiment lasted ~ 30 min (Fig. 1e).

#### Prerecorded videos

During filming, a male and a female lay still on a bed. The experimenter used a 90-cm-long stick with a white plastic ball (diameter 10 cm) attached to its end to deliver strokes to each model’s abdomen, left thigh, or right thigh. The duration of each stroke was 1 s, and each stroke covered ~ 20 cm of the model’s body. The time between the end of one touch and the onset of the next touch ranged between 3 and 5 s. The frequency of strokes was 12 times per minute. The order of strokes was pseudorandom (i.e., no more than two successive strokes of the same body part). Altogether, 36 strokes (12 to each body part) were delivered during each video. The videos were recorded with two identical cameras (GoPro HERO4 Silver, GoPro, Inc., San Mateo, CA, USA) placed parallel to each other (8 cm apart) just above the models’ heads. The recordings from both cameras were combined into a single frame using Final Cut Pro software (version 7, Apple Inc., Cupertino, CA, USA). Two versions of high-quality 3D videos were created: one for the male and one for the female body. Audio cues were then added to each video. These cues were either congruent with touches applied in the videos (same body parts, same onset, same duration) or delayed by 1 s. The experimenter listened to these cues during the experiment and applied touches accordingly. All other aspects were identical in the synchronous and asynchronous videos.

#### Knife threats

For each of the two videos, we recorded knife-threat events. During these events, a hand holding a knife entered the field of view from above and performed a stabbing movement toward the model’s body (Fig. 1c). The knife stopped just before hitting the body, changed direction (− 180°), and exited the field of view in the same way that it had entered. The whole event lasted 2 s. Great care was taken to ensure that the knife threats in the male and female versions of the videos looked as similar as possible. Knife threats in the synchronous and asynchronous versions of the same video (male or female) were identical. Subsequent knife threats within a given condition were also identical. After each knife threat, there was a 10 s pause when no strokes were delivered. In line with good ethical practice, before the experiment, we informed the participants about the knife threats in the videos to prevent overly high emotional stress.

#### Visuotactile stimulation during the experiment

The experimenter listened to audio cues from the videos (see earlier) and accordingly applied touches to the participant’s body. These cues were played via headphones, so the participants could not hear them. The number, order, type, length, velocity, and frequency of strokes during the experiment precisely followed the prerecorded videos (see earlier). To deliver touches, the experimenter used the same white ball attached to a stick that had been used in the video recordings.

#### Illusion questionnaires

Subjective experience of the full-body ownership illusion was quantified with a questionnaire that began with an open-ended sentence (“During the last session, there were times when…”). This sentence was followed by three illusion statements that quantified the explicit feeling of body ownership (I1; Fig. 1b) and the sensation of touch directly on the stranger’s body (I2 and I3; Fig. 1b). Ownership and referral of touch are considered to be the two core elements of the multisensory full-body illusion^25,26^. Apart from the illusion statements, the questionnaire included four control statements (C1–C4; Fig. 1b) that were added to control for potential task compliance or suggestibility effects. The questionnaire administered to the participants had items listed in the following pseudorandom order: C1, I1, C2, I2, C3, C4, I3. The participants marked their responses on a scale from − 3 (“strongly disagree”) to + 3 (“strongly agree”).

#### Skin conductance responses

The skin conductance response reflects increased sweating attributable to the activation of the autonomic nervous system^76^. When one’s own body is physically threatened, the threat triggers emotional feelings of fear and anticipation of pain that are associated with autonomic arousal. This arousal can be registered as a brief increase in skin conductance a few seconds after the threat event. Increased threat-evoked skin conductance responses, compared to a well-matched control condition, are often used as an index of body ownership in body illusion paradigms^24,30,38^. In the current experiment, data were recorded continuously with the Biopac system MP150 (Biopac Systems Inc., Goleta, CA, USA) and AcqKnowledge software (version 3.9). The following parameters were used: sampling rate = 100 Hz, low-pass filter = 1 Hz, high-pass filter = DC, gain = 5 μS/V, and CAL2 scale value = 5. Two Ag–AgCl electrodes (model TSD203, Biopac Systems Inc., Goleta, CA, USA) were placed on the volar surfaces of the distal phalanges of the participants’ left index and middle fingers. Isotonic paste (GEL101; Biopac Systems Inc., Goleta, CA, USA) was used to improve the skin contact and recording quality. At the beginning of the experiment, we asked the participants to take the deepest breath possible and hold it for a couple of seconds. In this way, we tested our equipment and established a near maximum response for each participant. The timing of threat events was marked in the recording file by the experimenter by pressing a laptop key immediately after the threat occurred.

#### Masculinity/femininity ratings

The participants marked their responses on a visual analog scale (Fig. 1d). Scale assignment was different for the male and female participants (Fig. 1d). Baseline ratings were generally greater than zero, as expected for a nontransgender group, but showed some degree of variability (mean = 2.22; SD = 0.97; min = − 1; max = 4).

### Experiment II

#### Participants

Sixty-four naïve adults participated (age: 27 ± 5; all right-handed; 32 females).

#### Procedure

The participants first completed a practice IAT (20 trials). The main study consisted of the same four conditions as those in Experiment I, that is, syncO, asyncO, syncS, and asyncS (Figs. 1a, 2a). After the initial phase of just watching the videos and feeling touches (30 s), the participants started the first IAT block (Fig. 2b,c). IAT stimuli were presented via headphones (Spectrum, Maxell Europe Ltd., Berkshire, UK). The participants used a wireless computer mouse held in the right hand to indicate responses. During each condition, the participants observed two “knife threats” (see further), one in the middle and one at the end of each condition (Fig. 2c). After each condition, the participants completed the same illusion questionnaire as in Experiment I (Fig. 1b). The order of the conditions was counterbalanced. The whole study lasted ~ 1 h (Fig. 2c).

#### Prerecorded videos

The videos were prepared analogously to those in Experiment I, but a different male and female were filmed to assure that our results were not driven by a certain body type or clothing style of the models (Fig. 2a). Strokes were applied to three body parts: abdomen, left thigh, and right thigh. The abdomen strokes were either single or double (1 s apart). The duration of each stroke was 1 s, and each stroke covered ~ 20 cm of the model’s body. The time between the offset of one touch and the onset of the next touch ranged from 3 to 6 s. The frequency of strokes was 12 times per minute. The touches were delivered in a pseudorandom sequence, with no more than three successive strokes on the same body part. Altogether, 88 touches (22 on each body part) were applied in each video. The videos were recorded with Infinity cameras (1080p Full HD, CamOneTec, Delbrück, Germany) and prepared in the same way as in Experiment I. In the synchronous videos, audio cues were matched with the touches applied in the videos, whereas in the asynchronous videos, the cues were delayed by 1 s and pertained to different body parts. Altogether, we created four versions (syncO, syncS, asyncO, asyncS) of the high-quality 3D videos, each lasting 7 min 5 s.

#### IAT

We used the auditory version of the brief gender identity IAT^47,77^. The instruction for one block was as follows: “The test will start in a few seconds. Please listen to the instructions. Try to go as fast as possible while making as few mistakes as possible. If the word belongs to the categories *female* or *self*, press left. If the word does not belong to these categories, press right. The test will begin now.” The instruction for the other block differed only with regard to category assignment: “If the word belongs to the categories *male* or *self*, press left. If the word does not belong to these categories, press right.” The key assignment remained the same for a given participant across all conditions but was counterbalanced between the participants. The order of IAT blocks was counterbalanced in the same way. The stimulus set consisted of twenty words (Fig. 2b) that were read by an English native speaker. The volume of each word sound was adjusted using Audacity software (the “normalize” effect; version 2.1.2, https://www.audacityteam.org). Each word was edited to have a duration similar to that of other words. Please note that the physical differences between stimuli cannot explain the main IAT results because the congruent and incongruent blocks used exactly the same stimuli. The participants had a maximum of 3 s to provide a response (time from the stimulus onset to the end of each trial). If no key was pressed within this time or the wrong key was pressed, the participants heard a “wrong” feedback beep. Each IAT block consisted of 60 trials (three repetitions of all 20 words) presented in random order. The procedure was self-paced, that is, the next trial started as soon as the participant responded in the previous trial (maximum duration of one block ~ 3 min). Presentation software (Neurobehavioral Systems Inc., Albany, CA, USA) was used to present the stimuli and record responses.

#### Knife threats

These events were recorded in the same way as in Experiment I (i.e., stabbing movement toward the abdomen; 2 s duration). We used triggers from the Presentation software to automatically flag the onset of the knife threats in the skin conductance recording files.

### Experiment III

#### Participants

Forty-five naïve adults participated (age: 26 ± 5; all right-handed; 22 females). One participant was excluded because he did not complete one of the questionnaires.

#### Procedure

The study lasted ~ 35 min and comprised two conditions: syncO and asyncO (Fig. 3a,b). Each condition lasted 14 min 10 s. After each condition, the participants filled out the illusion questionnaire (the same as in Experiments I and II) and the Bem Sex-Role Inventory; BSRI^49,50^ (see further). The order of conditions was counterbalanced across participants (Fig. 3b).

#### Prerecorded videos

The videos were prepared analogously to those in Experiments I and II. Four types of strokes (single abdomen, double abdomen, left thigh, and right thigh) were applied. The duration of each stroke was 1 s, and each stroke covered ~ 20 cm of the model’s body. The time between the offset of one touch and the onset of the next touch ranged from 2 to 10 s. The frequency of strokes was 12 times per minute. Different touches were delivered in a pseudorandom sequence, with no more than three successive strokes on the same body part. Altogether, 160 touches (40 on each body part) were applied in each video. Infinity cameras (1080p Full HD, CamOneTec, Delbrück, Germany) were used to record the videos. Audio cues were matched to touches in the synchronous videos and delayed by 1 s in the asynchronous videos.

#### BSRI

After each condition, the participants filled out a version of the BSRI^49,50^. The questionnaire contained 5 stereotypically masculine and 5 stereotypically feminine personality traits (Fig. 3c). Using a 7-point Likert scale (1—“not at all”; 7—“very much”), the participants rated how well each trait described them. Ten traits were rated after the first condition and the other ten after the second condition. The order of BSRI versions was counterbalanced.

### Analyses

#### Analysis of illusion questionnaires

For each participant and condition, we calculated “illusion scores” as the differences between the average illusion (I1–I3) and the control (C1–C4) ratings. To confirm successful induction of the illusion, we compared these illusion scores between the synchronous and asynchronous conditions. The results for individual questionnaire items are shown in Figs. S5 and S6. The effect of “ownership” used in the correlation analyses (Figs. 1h, 2f, 3f) was the difference between I1 ownership ratings in syncO–asyncO (one value per participant). The participants who experienced a strong body-sex-change illusion were selected using the median-split method applied to ownership scores (see above). The median-split analyses (Figs. 1i, 2g, and 3g) were performed mainly for display purposes and to complement the main analyses using continuous scores.

#### Analysis of skin conductance responses

Each response was measured as the difference between the maximum and minimum values during the 0–6 s period after each knife threat. Responses below 0.02 μS were treated as zeroes but were included in the analysis of the magnitude of skin conductance responses^76^. Statistical outliers were identified with the ± 1.5 interquartile criterion and removed from the dataset (16% and 6% of the values in Experiments I and II, respectively). Keeping the outliers did not change the main findings (main effect of synchrony in Experiment I: F_1,31_ = 5.76; *P* = 0.023; N = 32; Experiment II: F_1,63_ = 6.43; *P* = 0.014; N = 64; two-sided). We applied a square-root transformation to the skin conductance data^76^. Statistical models included the effect of “repetition”, which indicated how many knife threats had already occurred in the study (max. 12 in Experiment I and max. 8 in Experiment II). The magnitude of the skin conductance responses decreased exponentially with subsequent knife threats (Fig. S7). To “linearize” this relationship, we transformed the repetition number (1/repetition), which substantially improved the fit of the linear models to the data (Fig. S7; Experiment I: χ^2^ = 4.36; *P* < 0.005; Experiment II: χ^2^ = 37.26; *P* < 0.005; two-sided; N = 32 and N = 64, respectively). The effect of repetition (habituation) was highly significant (Tables S2 and S4), which was expected^76^. For the control analyses presented in Figs. S2 and S3, we (1) calculated residuals from the following model: SCR ~ repetition; (2) averaged them for a given participant and condition; and (3) calculated the difference: syncO–asyncO (Fig. S2) or syncS–asyncS (Fig. S3). Using the residuals accounted for the habituation effect (see earlier).

#### Analysis of masculinity/femininity ratings, IAT, and BSRI

Raw masculinity/femininity ratings were analyzed (n = 160; one value per condition). IAT data included only correct trials, in which reaction times were longer than 200 ms and shorter than 1,500 ms (95.5% of all trials; n = 29,147). Reaction times were log-transformed. The BSRI analysis was performed on raw ratings (n = 862; 18 ratings missing). Analyses of IAT and BSRI included random intercepts of “1|Item”, which accounted for possible variability between different words (Tables S4–S7).

#### Meta-analysis

For each participant in each experiment, we calculated the degree of gender identity updating. In Experiment I, this updating score was calculated as the following difference between the masculinity/femininity ratings: [(syncS + asyncS + asyncO)/3]–syncO. In Experiment II, this score was calculated as the difference between the average reaction times in each IAT block: [(syncS_i-c_ + asyncS_i-c_ + asyncO_i-c_)/3] – syncO_i-c_, where “i” and “c” denote “incongruent” and “congruent”, respectively. Finally, in Experiment III, the updating was calculated as the difference between average personality ratings from each condition: asyncO_c-i_–syncO_c-i_, where “c” and “i” correspond to stereotype-congruent and stereotype-incongruent traits, respectively. Because these scores were on different scales, we standardized them (i.e., from each participant’s score, we subtracted the group mean from the respective experiment and divided the result by the group standard deviation).

#### General statistical information

All statistical analyses were performed in RStudio and R software (version 3.3.3, The R Foundation for Statistical Computing, https://www.r-project.org). Linear mixed models were estimated using the “lme4” package. Information regarding model selection is provided in Table S1. All results are reported in Tables S2–S7. The distribution of residuals from each main model are shown in Fig. S8. *P*-values for the F-tests were based on Satterthwaite’s approximation to degrees of freedom, as implemented by the “lmerTest” package (Tables S2, S4, and S6). *P*-values for effect size coefficients (Tables S3, S5, and S6) and their 95% confidence intervals were obtained with the bootstrapping method by comparing a given coefficient value to its null distribution derived from resampling the original dataset (“boot” package; 1,000 simulations).

## Supplementary information

Supplementary information

Supplementary Movie S1

Supplementary Movie S2

## Data Availability

We do not have ethics approval to make the raw data from individual subjects publicly available.
