# Supplementary material for: Fluidity of gender identity induced by illusory body-sex change
Source: Sci Rep. 2020 Sep 1;10:14385. doi: 10.1038/s41598-020-71467-z (PMC7463009; doi:10.1038/s41598-020-71467-z)
Supplement: Supplementary file 1 — Supplementary information [file 41598_2020_71467_MOESM1_ESM.pdf]

Supplementary Information

Fluidity of gender identity induced by illusory body-sex change

P. Tacikowski, J. Fust, and H. H. Ehrsson

Correspondence to pawel.tacikowski@ki.se

This document includes the following:

Supplementary Figures S1-S8

Supplementary Tables S1-S7

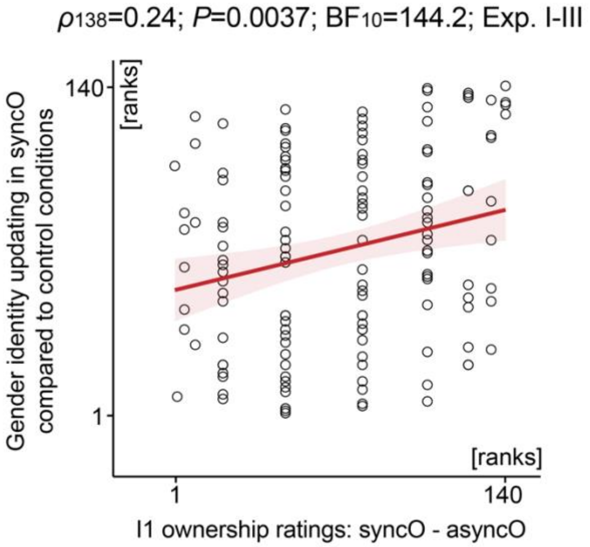

Fig. S1. Meta-analysis of data from all three experiments confirmed that illusory ownership of the opposite-sex body was associated with increased updating of the sense of own gender (Spearman's correlation; two-sided; N=140; BF_{10} indicates a Bayes factor in support of the alternative hypothesis).

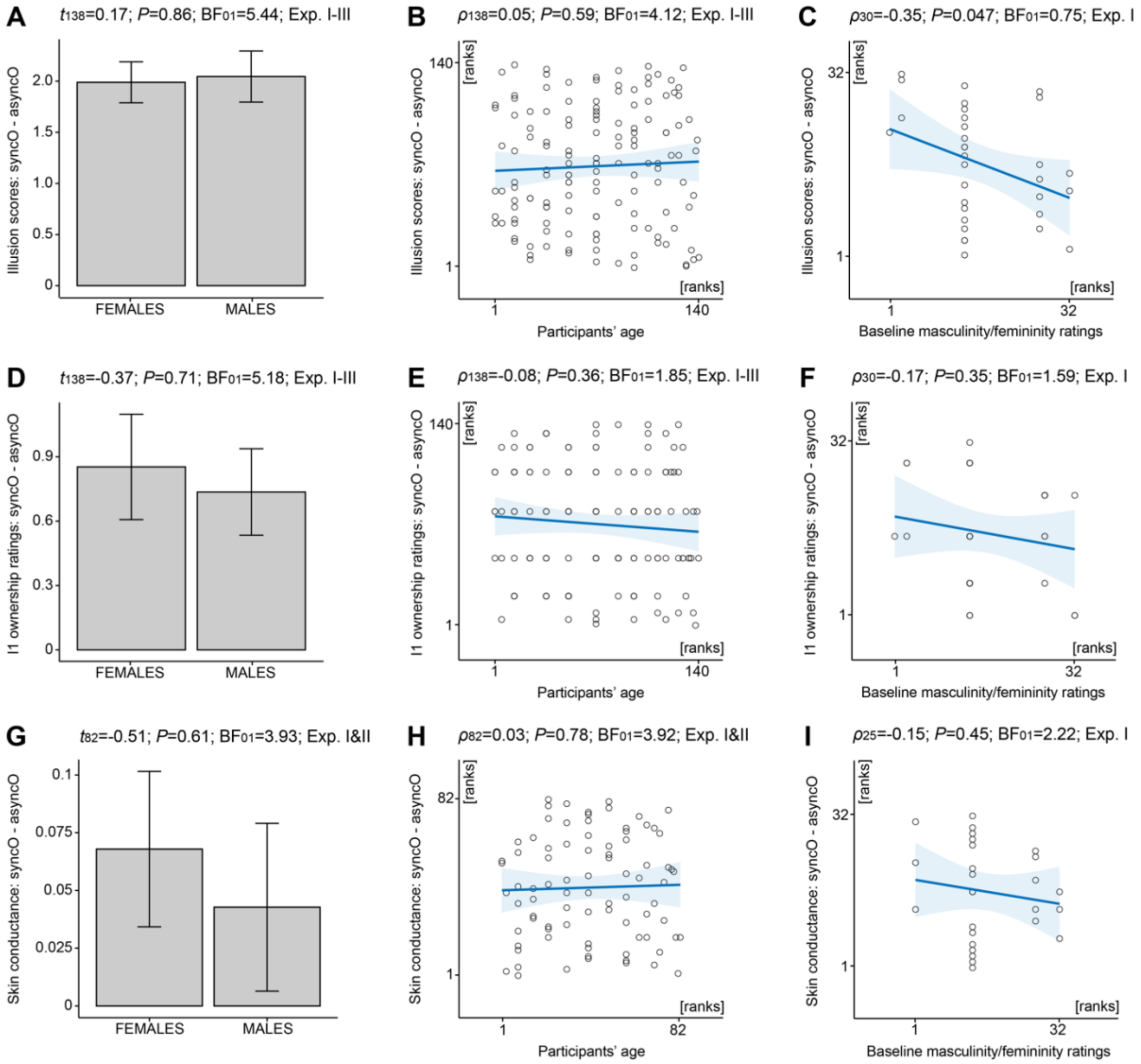

Fig. S2. Males and females experienced the body-sex-change illusion in syncO with similar strength, and there was no significant relationship between the strength of the illusion and the participants' age or baseline masculinity/femininity ratings. The strength of the body-sex-change illusion was measured as the syncO – asyncO difference between illusion scores **(A-C)**, I1 ownership ratings **(D-F)**, and skin conductance responses **(G-I)**. Continuous variables were analyzed with the Spearman's correlation test. The effect of the participants' sex was tested with independent-samples t-tests. BF_{01} indicates Bayes factors in support of the null hypotheses. All *P*-values are two-sided. Bar plots show means±*SE*.

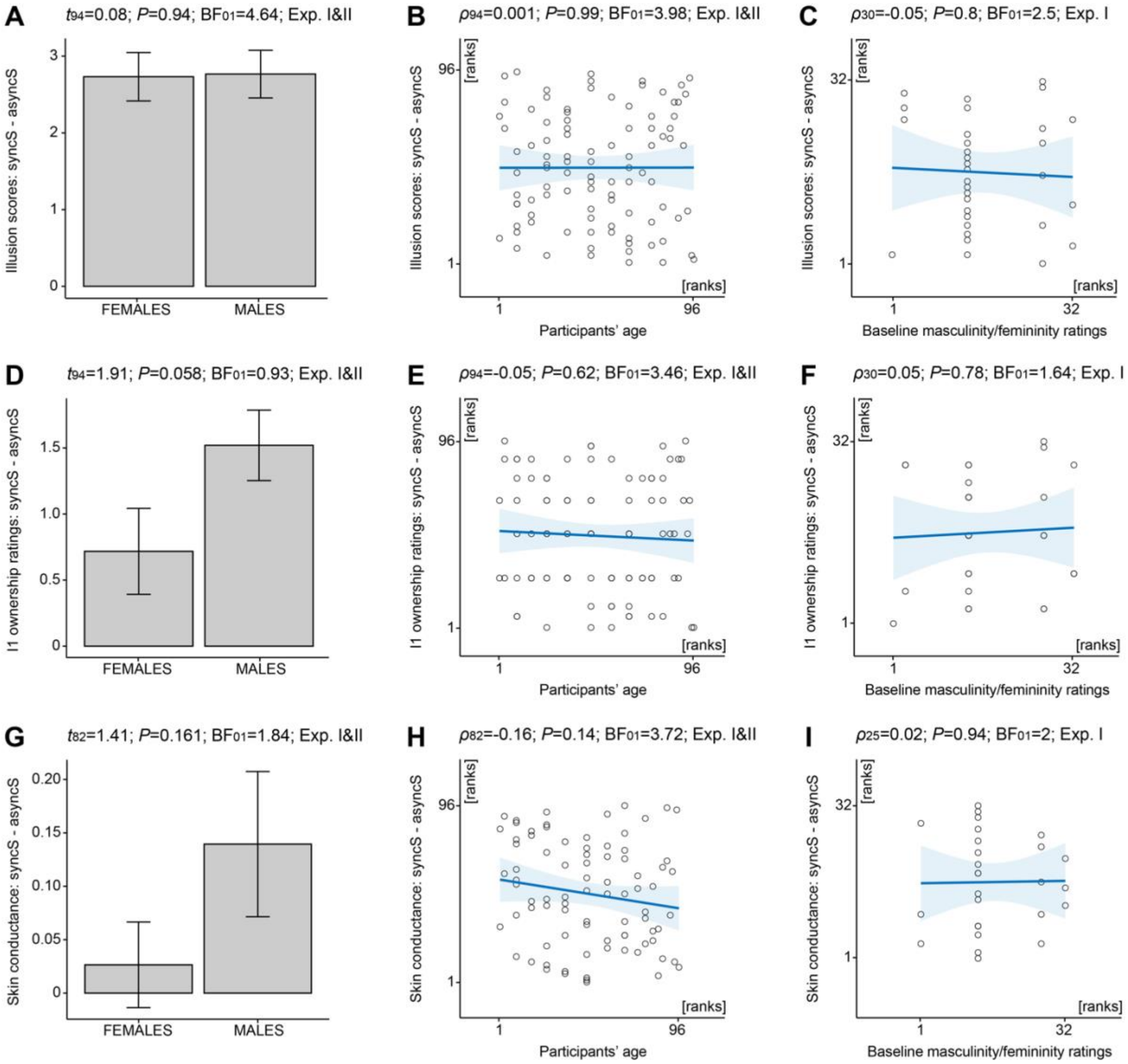

Fig. S3. The strength of illusory ownership of the same-sex stranger's body in syncS did not differ significantly between males and females, and there was no significant relationship between the strength of the illusion and the participants' age or baseline masculinity/femininity ratings. The illusion in syncS was measured as the syncS – asyncS difference between illusion scores **(A-C)**, I1 ownership ratings **(D-F)**, and skin conductance responses **(G-I)**. Continuous variables were analyzed with the Spearman's correlation test. The effect of the participants' sex was tested with independent-samples t-tests. BF_{01} indicates Bayes factors in support of the null hypotheses. All *P*-values are two-sided. Bar plots show means±*SE.*

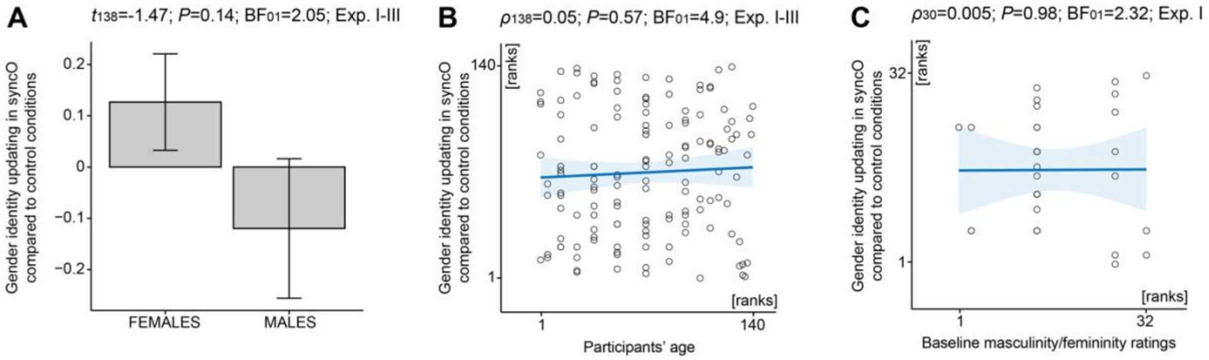

Fig. S4. The degree of gender identity updating did not significantly differ between males and females **(A)** and was not significantly modulated by the participants' age **(B)** or baseline masculinity/femininity ratings **(C)**. Age and masculinity/femininity ratings were analyzed with the Spearman's correlation test. The effect of the participants' sex was tested with the independent-samples t-test. BF_{01} indicates the Bayes factors in support of the null hypothesis. All *P*-values are two-sided. Bar plots show means±*SE*.

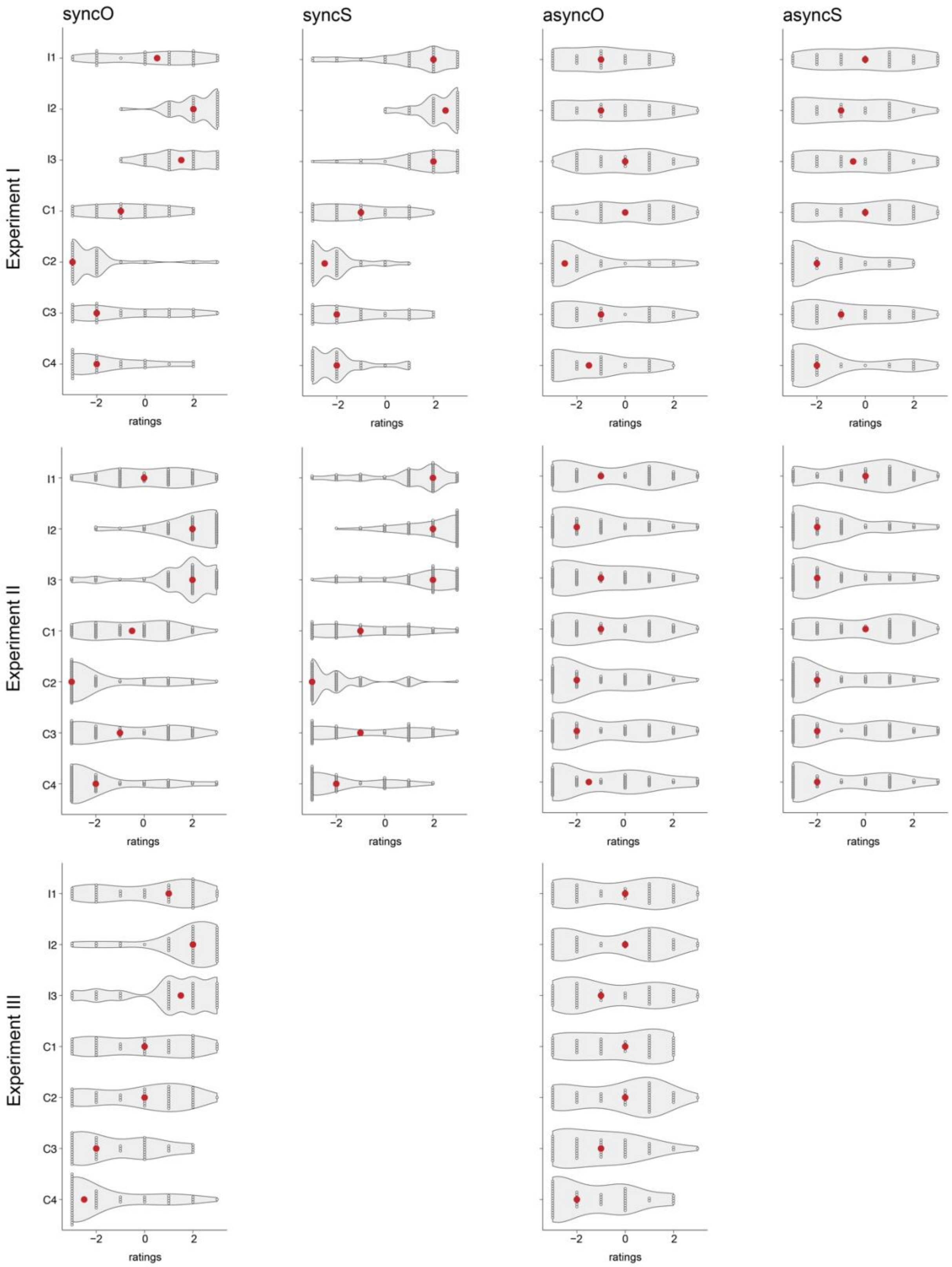

Fig. S5. Illusion questionnaire items. Red dots represent medians. Small white dots are individual ratings. “Clouds” are probability densities of ratings at different values. I1:I3 are illusion items. C1:C4 are control items.

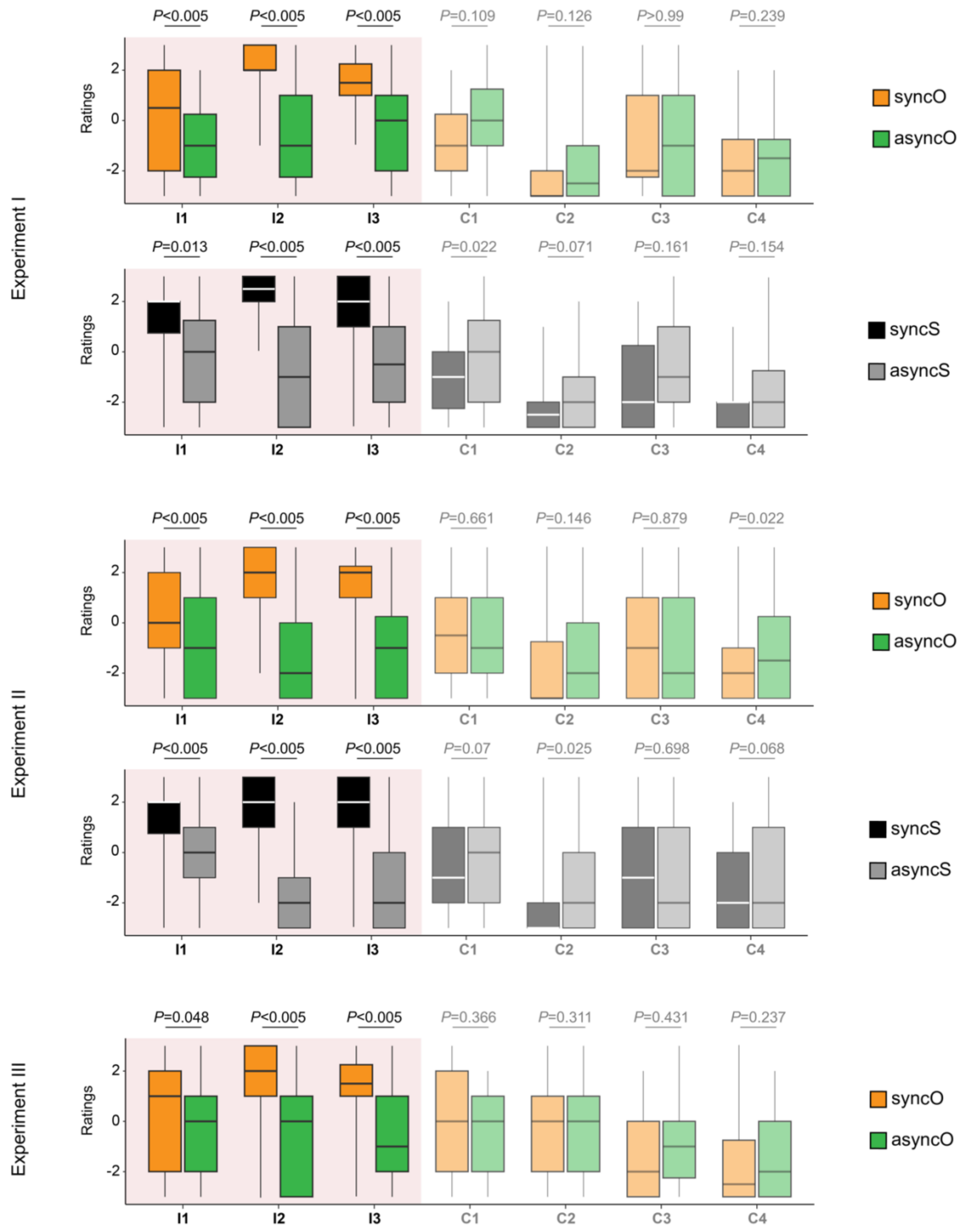

Fig. S6. Results for illusion questionnaire items. The lower and upper hinges of each boxplot correspond to the first and third quartiles, respectively (i.e., interquartile ranges; IQR). The horizontal lines are the medians. The upper and lower whiskers correspond to the maximum and minimum values. For pairwise comparisons, we used the nonparametric Wilcoxon signed-rank tests (two-sided). I1:I3 are illusion items. C1:C4 are control items. Please note that, in rare instances where control ratings significantly differed, the medians were lower in the synchronous than in the asynchronous condition.

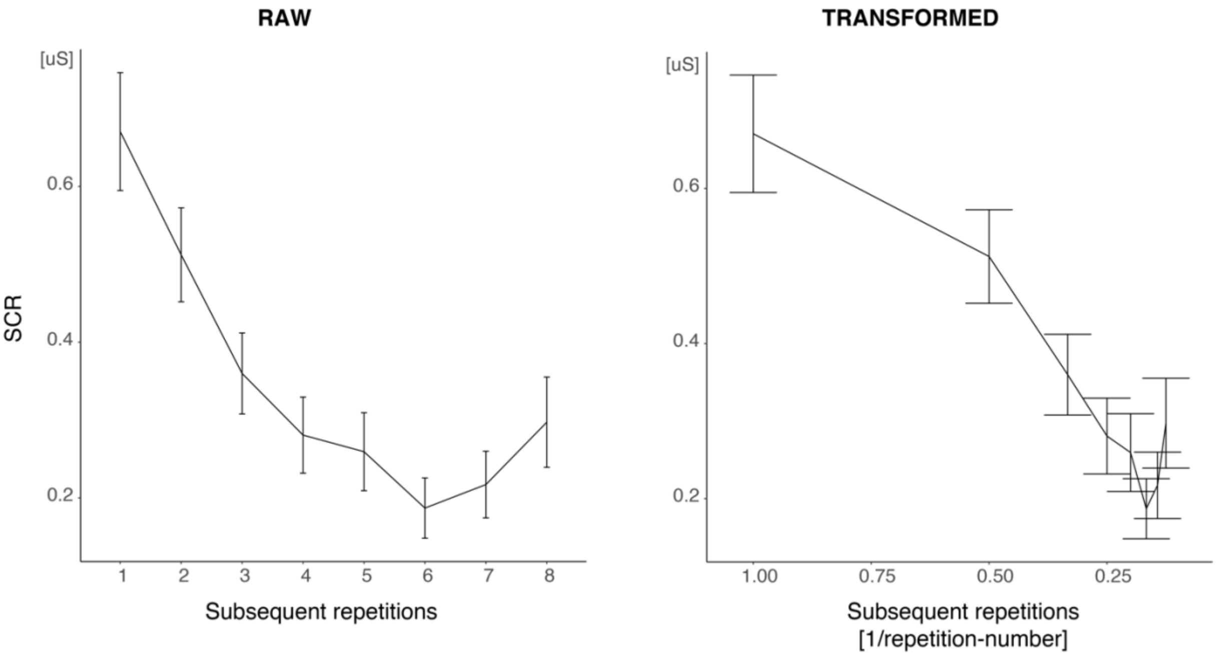

Fig. S7. Transformation of the repetition number improved the analysis of skin conductance responses (SCRs). Plots show the magnitude of SCRs in Experiment II averaged across all conditions (data from Experiment I looked analogous). Applying the 1/repetition transformation "linearized" the relationship between SCRs and the repetition number, which substantially improved the fit of the linear mixed model to the data (see Methods).

Table S1. Model selection in Experiments I-III^.

**Exp.**	**Meas.**	**Full model**	**df**	**AIC**
Exp. I	Ill. S.	Score ~ Sync×Body + (Sync + Body|ID)	11	474
	SCR	SCR ~ Sync×Body×Rep + (Sync + Body + Rep|ID)	19	-207
	M/F	Rating ~ Sync×Body + (Sync + Body|ID)	11	438
		Rating ~ Sync×Body×Own + (Sync + Body|ID)	15	434
	M/F'	Rating ~ Sync×Body + (Sync + Body|ID)	11	152
Exp. II	Ill. S.	Score ~ Sync×Body + (Sync + Body|ID)	11	961
	SCR	SCR ~ Sync×Body×Rep + (Sync + Body + Rep|ID)	19	62
	IAT	RT ~ Sync×Body×Block + (Sync + Body + Block|ID) + (1|Item)	20	-55144
		RT ~ Sync×Body×Block×Own + (Sync + Body + Block|ID) + (1|Item)	28	-55173
	IAT'	RT ~ Sync×Body×Block + (Sync + Body + Block|ID) + (1|Item)	20	-19334
Exp. III	Ill. S.	Score ~ Sync + (1|ID)	4	336
	BSRI	Rating ~ Sync×Cong + (1|ID) + (1|Item)	7	2764
		Rating ~ Sync×Cong×Own + (Sync + Cong|ID) + (1|Item)	16	2708
	BSRI'	Rating ~ Sync×Cong + (1|ID) + (1|Item)	7	1318
**Exp.**	**Meas.**	**Selected model**	**df**	**AIC**
Exp. I	Ill. S.	Score ~ Sync + Body + (Sync|ID)	7	469
	SCR	SCR ~ Sync + Body×Rep + (Sync + Body + Rep|ID)	16	-212
	M/F	Rating ~ Sync×Body + (Sync + Body|ID)	11	438
		Rating ~ Sync×Body×Own + (Sync + Body|ID)	15	434
	M/F'	Rating ~ Sync×Body + (Body|ID)	8	152
Exp. II	Ill. S.	Score ~ Sync + (Sync + Body|ID)	9	961
	SCR	SCR ~ Sync + Rep + (Sync + Rep|ID)	10	49
	IAT	RT ~ Sync×Body + Cong + (Sync + Body + Cong|ID) + (1|Item)	17	-55146
		RT ~ Sync×Body×Block×Own + (Sync + Body + Block|ID) + (1|Item)	28	-55173
	IAT'	RT ~ Sync×Body×Block + (Sync + Body + Block|ID) + (1|Item)	20	-19334
Exp. III	Ill. S.	Score ~ Sync + (1|ID)	4	336
	BSRI	Rating ~ Cong + (1|ID) + (1|Item)	5	2761
		Rating ~ Sync×Cong×Own + (Cong|ID) + (1|Item)	13	2707
	BSRI'	Rating ~ Sync×Cong + (1|ID) + (1|Item)	7	1318

^ – Model selection was performed with the "lmerTest" package ("step" function). The full models were maximally complex, with the following restrictions: (i) they had to be "nested" and (ii) they had to converge. Please note that the "Own" factor (I1-ownership ratings: syncO – asyncO) consisted of one value per participant; thus, this factor was not used as a random effect with the grouping factor "ID". All models included fixed and random intercepts. Hereafter, models including complex interactions also included simpler interactions and main effects, for example, "Sync×Body×Own" is equivalent to "1 + Sync + Body + Own + Sync×Body + Sync×Own + Body×Own + Sync×Body×Own," whereas "(Cong|ID)" is equivalent to "(1 + Cong|ID).
' – Analysis performed on a subset of participants who indicated a strong illusion in syncO (see Methods).
Abbreviations in alphabetical order: **AIC** – Akaike information criterion; **Body** – factor with two levels: same-sex vs. opposite-sex; **BSRI** – Bem Sex-Role Inventory; **Cong** – factor with two levels: congruent vs. incongruent; **df** – degrees of freedom; **Exp.** – experiment; **IAT** – Implicit Association Test; **ID** – participants; **Ill. S.** – illusion scores (illusion questionnaire ratings: (I1+I2+I3)/3 – (C1+C2+C3+C4)/4); **Item** – words in IAT or BSRI; **M/F** – masculinity/femininity ratings; **Meas.** – measure; **Own** – I1-ownership ratings: syncO-asyncO; **Rep** – SCR repetition number; **RT** – reaction times; **SCR** – skin-conductance responses; **Sync** – factor with two levels: synchronous vs. asynchronous.

Table S2. Results from Experiment I^.

Meas.	Model	Effect	dfN	dfD	F	P
Ill. S.	Score ~ Sync + Body + (Sync|ID)	Sync	1	32	64.48	<0.005
		Body	1	64	5.28	0.025
SCR	SCR ~ Sync + Body×Rep + (Sync + Body + Rep|ID)	Sync	1	27	10.98	<0.005
		Rep	1	14	31.09	<0.005
		Body×Rep	1	76	5.60	0.020
M/F	Rating ~ Sync×Body + (Sync + Body|ID)	Sync×Body	1	32	10.12	<0.005
	Rating ~ Sync×Body×Own + (Sync + Body|ID)	Sync×Body×Own	1	32	8.05	0.008
M/F’	Rating ~ Sync×Body + (Body|ID)	Sync×Body	1	24	18.86	<0.005

^ – For brevity, only significant effects and interactions are reported. If a complex interaction was significant, main effects and simpler interactions were skipped.
’ – Analysis performed on a subset of participants who indicated a strong illusion in syncO (see Methods).
Abbreviations in alphabetical order: **Body** – factor with two levels: same-sex vs. opposite-sex; **dfN** – degrees of freedom in the numerator; **dfD** – degrees of freedom in the denominator; **F** – F-ratio; **ID** – participants; **Ill. S.** – illusion scores (illusion questionnaire ratings: (I1+I2+I3)/3 – (C1+C2+C3+C4)/4); **M/F** – masculinity/femininity ratings; **Meas.** – measure; **Own** – I1-ownership ratings: syncO-asyncO; **P** – p-values based on Satterthwaite’s approximation to degrees of freedom; **Rep** – SCR repetition number; **SCR** – skin-conductance responses; **Sync** – factor with two levels: synchronous vs. asynchronous.

Table S3. Effect sizes in Experiment I^.

Meas.	Model	Effect	b	SE	df	t	P	CI-l	CI-u
Ill. S.	Opp: Score ~ Sync + (1|ID)	Sync(syncO)	2.2	0.3	32	7.02	<0.005	1.6	2.8
	Same: Score ~ Sync + (1|ID)	Sync(syncS)	2.7	0.4	32	6.87	<0.005	1.9	3.5
SCR	Opp: SCR ~ Sync + (1|ID)	Sync(syncO)	0.05	0.02	122	2.46	0.010	0.01	0.09
	Same: SCR ~ Sync + (1|ID)	Sync(syncS)	0.11	0.02	136	4.30	<0.005	0.06	0.15
M/F	syncO vs. baseline: Rating ~ Cond + (1|ID)	Cond(syncO)	-2.2	0.4	32	-6.22	<0.005	-2.9	-1.5
	syncO vs. syncS: Rating ~ Cond + (1|ID)	Cond(syncO)	-2.2	0.4	32	-6.06	<0.005	-2.9	-1.5
	syncO vs. asyncS: Rating ~ Cond + (1|ID)	Cond(syncO)	-1.8	0.4	32	-4.83	<0.005	-2.5	-1.0
	syncO vs. asyncO: Rating ~ Cond + (1|ID)	Cond(syncO)	-0.4	0.2	32	-1.87	0.060	-0.9	0.0
M/F	syncO: Rating ~ Own	Own	-0.6	0.2	30	-2.29	0.022	-1.1	-0.1
	syncS: Rating ~ Own	Own	0.1	0.2	30	0.41	0.669	-0.2	0.4
	asyncO: Rating ~ Own	Own	-0.2	0.2	30	-0.88	0.403	-0.6	0.2
	asyncS: Rating ~ Own	Own	-0.1	0.2	30	-0.38	0.724	-0.4	0.3
	baseline: Rating ~ Own	Own	-0.1	0.1	30	-1.07	0.222	-0.3	0.1
M/F’	syncO vs. baseline: Rating ~ Cond + (1|ID)	Cond(syncO)	-3.2	0.5	24	-6.14	<0.005	-4.2	-2.2
	syncO vs. syncS: Rating ~ Cond + (1|ID)	Cond(syncO)	-3.4	0.5	24	-6.60	<0.005	-4.5	-2.4
	syncO vs. asyncS: Rating ~ Cond + (1|ID)	Cond(syncO)	-2.9	0.6	24	-5.02	<0.005	-4.1	-1.7
	syncO vs. asyncO: Rating ~ Cond + (1|ID)	Cond(syncO)	-1.3	0.36	12	-3.70	<0.005	-2.0	-0.6

^ – Units of “b”, “SE”, “CI-l”, and “CI-u” are M/F ratings.
’ – Analysis performed on a subset of participants who indicated a strong illusion in syncO (see Methods).
Abbreviations in alphabetical order: **asyncO** – asynchronous opposite-sex condition; **asyncS** – asynchronous same-sex condition; **b** – coefficient; **CI-l** and **CI-u** – lower and upper boundaries of the 95% confidence interval, respectively (bootstrapping method; 1000 simulations; “boot” package); **Cond** – condition; **df** – degrees of freedom; **ID** – participants; **Ill. S.** – illusion scores (illusion questionnaire ratings: (I1+I2+I3)/3 – (C1+C2+C3+C4)/4); **M/F** – masculinity/femininity ratings; **Meas.** – measure; **Own** – I1-ownership ratings: syncO-asyncO; **Opp** – opposite-sex; **P** – p-values (bootstrapping method; 1000 simulations; “boot” package); **Same** – same-sex; **SCR** – skin-conductance responses; **Sync** – factor with two levels: synchronous vs. asynchronous; **SE** – standard error; **syncO** – synchronous opposite-sex condition; **syncS** – synchronous same-sex condition; **t** – t-test statistic.

Table S4. Results from Experiment II^.

Meas.	Model	Effect	dfN	dfD	F	P
Ill. S.	Score ~ Sync + (Sync + Body|ID)	Sync	1	64	125.65	<0.005
SCR	SCR ~ Sync + Rep + (Sync + Rep|ID)	Sync	1	60	4.97	0.03
		Rep	1	54	104.42	<0.005
IAT	RT ~ Sync×Body + Cong + (Sync + Body + Cong|ID) + (1|Item)	Sync	1	64	0.28	0.601
		Cong	1	64	18.89	<0.005
		Sync×Body	1	28877	6.30	0.012
IAT	RT ~ Sync×Body×Cong×Own + (Sync + Body + Cong|ID) + (1|Item)	Sync×Body×Cong×Own	1	28878	17.03	<0.005
	syncO: RT ~ Cong×Own + (1|ID) + (1|Item)	Cong*Own	1	7207	9.37	<0.005
	syncS: RT ~ Cong×Own + (1|ID) + (1|Item)	Cong*Own	1	7213	14.66	<0.005#
	asyncO: RT ~ Cong×Own + (1|ID) + (1|Item)	Cong*Own	1	7223	1.08	0.299
	asyncS: RT ~ Cong×Own + (1|ID) + (1|Item)	Cong*Own	1	7173	0.19	0.666
IAT’	RT ~ Sync×Body×Cong + (Sync + Body + Cong|ID) + (1|Item)	Sync*Body*Cong	1	10793	4.04	0.045

^ – For brevity, only significant effects and interactions are reported. If a complex interaction was significant, main effects and simpler interactions were skipped.
# – Please note that the interaction in syncS was in the opposite direction than in syncO (Table S5).
’ – Analysis performed on a subset of participants who indicated a strong illusion in syncO (see Methods).
Abbreviations in alphabetical order: **asyncO** – asynchronous opposite-sex condition; **asyncS** – asynchronous same-sex condition; **Body** – factor with two levels: same-sex vs. opposite-sex; **Cong** – factor with two levels: congruent vs. incongruent; **dfN** – degrees of freedom in the numerator; **dfD** – degrees of freedom in the denominator; **F** – F-ratio; **IAT** – Implicit Association Test; **ID** – participants; **Ill. S.** – illusion scores (illusion questionnaire ratings: (I1+I2+I3)/3 – (C1+C2+C3+C4)/4); **Item** – words in IAT; **Meas.** – measure; **Own** – I1-ownership ratings: syncO-asyncO; **P** – p-values based on Satterthwaite’s approximation to degrees of freedom; **RT** – reaction times; **SCR** – skin-conductance responses; **Sync** – factor with two levels: synchronous vs. asynchronous; **syncO** – synchronous opposite-sex condition; **syncS** – synchronous same-sex condition.

Table S5. Effect sizes in Experiment II^.

Meas.	Model	Effect	b	SE	df	t	P	CI-l	CI-u
Ill. S.	Opp: Score ~ Sync + (1|ID)	Sync(syncO)	2.3	0.26	64	9.08	<0005	1.8	2.8
	Same: Score ~ Sync + (1|ID)	Sync(syncS)	2.8	0.26	64	10.54	<0005	2.2	3.3
SCR	Opp: SCR ~ Sync + (1|ID)	Sync(syncO)	0.09	0.04	177	2.31	0.018	0.02	0.16
	Same: SCR ~ Sync + (1|ID)	Sync(syncS)	0.09	0.05	175	1.90	0.058	-0.01	0.17
IAT	syncO: RT ~ Cong + (1|ID) + (1|Item)	Cong(cong)	-18	4	7208	-4.85	<0005	-27	-11
	syncS: RT ~ Cong + (1|ID) + (1|Item)	Cong(cong)	-28	4	7213	-7.35	<0005	-36	-21
	asyncO: RT ~ Cong + (1|ID) + (1|Item)	Cong(cong)	-25	4	7224	-6.68	<0005	-32	-18
	asyncS: RT ~ Cong + (1|ID) + (1|Item)	Cong(cong)	-25	4	7174	-6.51	<0005	-33	-17
IAT	syncO-cong: RT ~ Own + (1|Item)	Own	6	1	3636	4.33	<0005	3	9
	syncO-incong: RT ~ Own + (1|Item)	Own	0	1	3616	0.25	0.812	-3	3
	syncS-cong: RT ~ Own + (1|Item)	Own	-2	1	3673	-1.70	0.082	-5	0
	syncS-incong: RT ~ Own + (1|Item)	Own	4	2	3584	2.54	0.008	1	7
	asyncO-cong: RT ~ Own + (1|Item)	Own	-2	1	3655	-1.73	0.088	-5	0
	asyncO-incong: RT ~ Own + (1|Item)	Own	-1	2	3613	-0.77	0.446	-4	2
	asyncS-cong: RT ~ Own + (1|Item)	Own	2	1	3642	1.14	0.260	-1	5
	asyncS-incong: RT ~ Own + (1|Item)	Own	1	2	3574	0.97	0.352	-2	4
IAT’	syncO: RT ~ Cong + (1|ID) + (1|Item)	Cong(cong)	-7	7	2657	-1.03	0.250	-20	6
	syncS: RT ~ Cong + (1|ID) + (1|Item)	Cong(cong)	-39	7	2696	-5.90	<0005	-52	-26
	asyncO: RT ~ Cong + (1|ID) + (1|Item)	Cong(cong)	-25	6	2713	-3.88	<0005	-36	-12
	asyncS: RT ~ Cong + (1|ID) + (1|Item)	Cong(cong)	-30	7	2670	-4.62	<0005	-44	-18

^ – Units of “b”, “SE”, “CI-l”, and “CI-u” are milliseconds.
’ – Analysis performed on a subset of participants who indicated a strong illusion in syncO (see Methods).
Abbreviations in alphabetical order: **asyncO** – asynchronous opposite-sex condition; **asyncS** – asynchronous same-sex condition; **b** –coefficient; **CI-l** and **CI-u** – lower and upper boundaries of the 95% confidence interval, respectively (bootstrapping method; 1000 simulations; “boot” package); **Cong** – factor with two levels: congruent (**cong**) vs. incongruent (**incong**); **df** – degrees of freedom; **ID** – participants; **Ill. S.** – illusion scores (illusion questionnaire ratings: (I1+I2+I3)/3 – (C1+C2+C3+C4)/4); **Item** – words in IAT; **Meas.** – measure; **Own** – I1-ownership ratings: syncO-asyncO; **Opp** – opposite-sex; **P** – p-values (bootstrapping method; 1000 simulations; “boot” package); **RT** – reaction times; **Same** – same-sex; **SCR** – skin-conductance responses; **Sync** – factor with two levels: synchronous vs. asynchronous; **SE** – standard error; **syncO** – synchronous opposite-sex condition; **syncS** – synchronous same-sex condition; **t** – t-test statistic.

Table S6. Results from Experiment III^.

Meas.	Model	Effect	dfN	dfD	F	P
Ill. S.	Score ~ Sync + (1|ID)	Sync	1	44	35.88	<0.005
BSRI	Rating ~ Cong + (1|ID) + (1|Item)	Cong	1	800	4.86	0.028
BSRI	Rating ~ Sync×Cong×Own + (Cong|ID) + (1|Item)	Sync×Cong×Own	1	759	5.60	0.018
	syncO: Rating ~ Cong×Own + (1|ID) + (1|Item)	Cong×Own	1	374	13.46	<0.005
	asyncO: Rating ~ Cong×Own + (1|ID) + (1|Item)	Cong×Own	1	368	0.23	0.630
BSRI'	Rating ~ Sync×Cong + (1|ID) + (1|Item)	Sync*Cong	1	357	1.75	0.186

^ – For brevity, only significant effects and interactions are reported. If a complex interaction was significant, main effects and simpler interactions were skipped.
' – Analysis performed on a subset of participants who indicated a strong illusion in syncO (see Methods).
Abbreviations in alphabetical order: **asyncO** – asynchronous opposite-sex condition; **Body** – factor with two levels: same-sex vs. opposite-sex; **BSRI** – Bem Sex-Role Inventory; **Cong** – factor with two levels: congruent vs. incongruent; **dfN** – degrees of freedom in the numerator; **dfD** – degrees of freedom in the denominator; **F** – F-ratio; **ID** – participants; **Ill. S.** – illusion scores (illusion questionnaire ratings: (I1+I2+I3)/3 – (C1+C2+C3+C4)/4); **Item** – traits in BSRI; **Meas.** – measure; **Own** – I1-ownership ratings: syncO-asyncO; **P** – p-values based on Satterthwaite's approximation to degrees of freedom; **Sync** – factor with two levels: synchronous vs. asynchronous; **syncO** – synchronous opposite-sex condition.

Table S7. Effect sizes in Experiment III^.

Meas.	Model	Effect	b	SE	df	t	P	CI-l	CI-u
Ill. S.	Opp: Score ~ Sync + (1|ID)	Sync(syncO)	1.5	0.2	44	5.99	<0.005	1.0	2.0
BSRI	Rating ~ Cong + (1|ID) + (1|Item)	Cong(cong)	0.17	0.08	800	2.21	0.026	0.02	0.33
	syncO-cong: Rating ~ Own + (1|Item)	Own	-0.07	0.05	200	-1.64	0.090	0.17	0.01
	syncO-incong: Rating ~ Own + (1|Item)	Own	0.14	0.04	199	3.23	<0.005	0.05	0.22
	asyncO-cong: Rating ~ Own + (1|Item)	Own	-0.01	0.05	194	-0.14	0.914	-0.09	0.09
	asyncO-incong: Rating ~ Own + (1|Item)	Own	0.03	0.05	198	0.55	0.596	-0.08	0.13
BSRI'	syncO: Rating ~ Cong + (1|ID) + (1|Item)	Cong(cong)	0.05	0.17	160	0.30	0.796	-0.28	0.39
	asyncO: Rating ~ Cong + (1|ID) + (1|Item)	Cong(cong)	0.36	0.17	160	2.19	0.020	0.05	0.70

^ – Units of "b", "SE", "CI-l", and "CI-u" correspond to BSRI ratings.
' – Analysis performed on a subset of participants who indicated a strong illusion in syncO (see Methods).
Abbreviations in alphabetical order: **asyncO** – asynchronous opposite-sex condition; **b** –coefficient; **BSRI** – Bem Sex-Role Inventory; **CI-l** and **CI-u** – lower and upper boundaries of the 95% confidence interval, respectively (bootstrapping method; 1000 simulations; "boot" package); **Cong** – factor with two levels: congruent (**cong**) vs. incongruent (**incong**); **df** – degrees of freedom; **ID** – participants; **Ill. S.** – illusion scores (illusion questionnaire ratings: (I1+I2+I3)/3 – (C1+C2+C3+C4)/4); **Item** – traits in BSRI; **Meas.** – measure; **Own** – I1-ownership ratings: syncO-asyncO; **Opp** – opposite-sex; **P** – p-values (bootstrapping method; 1000 simulations; "boot" package); **SE** – standard error; **Sync** – factor with two levels: synchronous vs. asynchronous; **syncO** – synchronous opposite-sex condition; **t** – t-test statistic.

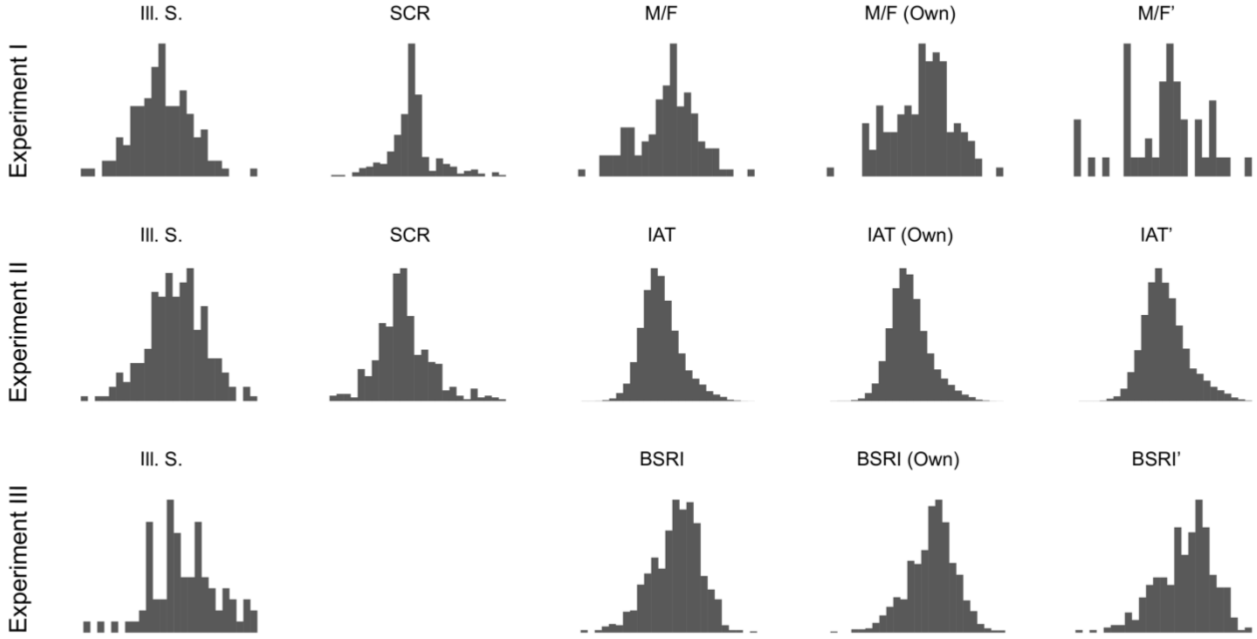

Fig. S8. Distributions of residuals. Plots show histograms of residuals from all main models used in the study (see Tables S2, S5, and S7). All distributions are approximately normally distributed. Please note that linear mixed models are typically reliable even when the normality assumption is strongly violated[1]. Additionally, to provide robust estimates of effect sizes, we performed bootstrapping tests (Tables S3, S5, and S7). Abbreviations in alphabetical order: **BSRI** – Bem Sex-Role Inventory; **IAT** – Implicit Association Test; **Ill. S.** – illusion scores (illusion questionnaire ratings: (I1+I2+I3)/3 – (C1+C2+C3+C4)/4); **M/F** – masculinity/femininity ratings; **Own** – I1-ownership ratings: syncO-asyncO; **SCR** – skin-conductance responses. ' – Analysis performed on a subset of participants who indicated a strong illusion in syncO (see Methods).

Reference:

1. Jacqmin-Gadda, H., Sibillot, S., Proust, C., Molina, J. M. & Thiébaut, R. Robustness of the linear mixed model to misspecified error distribution. *Comput. Stat. Data Anal.* **51**, 5142–5154 (2007).
